# Physiologically Based Pharmacokinetic Modeling Is Essential in ^90^Y-Labeled Anti-CD66 Radioimmunotherapy

**DOI:** 10.1371/journal.pone.0127934

**Published:** 2015-05-26

**Authors:** Peter Kletting, Christian Maaß, Sven Reske, Ambros J. Beer, Gerhard Glatting

**Affiliations:** 1 Department of Nuclear Medicine, Ulm University, 89070, Ulm, Germany; 2 Medical Radiation Physics/Radiation Protection, Medical Faculty Mannheim, Heidelberg University, 68167, Mannheim, Germany; Genentech, UNITED STATES

## Abstract

**Introduction:**

Radioimmunotherapy (RIT) with ^90^Y-labeled anti-CD66 antibody is used to selectively irradiate the red marrow (RM) before blood stem cell transplantation of acute leukemia patients. To calculate the activity to administer, time-integrated activity coefficients are required. These are estimated prior to therapy using gamma camera and serum measurements after injection of ^111^In labeled anti-CD66 antibody. Equal pre-therapeutic and therapeutic biodistributions are usually assumed to calculate the coefficients. However, additional measurements during therapy had shown that this assumption had to be abandoned. A physiologically based pharmacokinetic (PBPK) model was developed to allow the prediction of therapeutic time-integrated activity coefficients in eight patients.

**Aims:**

The aims of the study were to demonstrate using a larger patient group 1) the need to perform patient-specific dosimetry in ^90^Y-labeled anti-CD66 RIT, 2) that pre-therapeutic and therapeutic biodistributions differ, and most importantly 3) that this difference in biodistributions can be accurately predicted using a refined model.

**Materials and Methods:**

Two new PBPK models were developed considering fully, half and non-immunoreactive antibodies and constraints for estimating the RM antigen number. Both models were fitted to gamma camera and serum measurements of 27 patients. Akaike weights were used for model averaging. Time-integrated activity coefficients for total body, liver, spleen, RM and serum were calculated. Model-based predictions of the serum biokinetics during therapy were compared to actual measurements.

**Results:**

Variability of the RM time-integrated activity coefficients ((37.3±7.5) h) indicates the need for patient-specific dosimetry. The relative differences between pre-therapeutic and therapeutic serum time-activity curves were (-25±16)%. The prediction accuracy of these differences using the refined PBPK models was (-3±20)%.

**Conclusion:**

Individual treatment is needed due to biological differences between patients in RIT with ^90^Y-labeled anti-CD66 antibody. Differences in pre-therapeutic and therapeutic biokinetics are predominantly caused by different degrees of saturation due to different amounts of administered antibody. These differences could be predicted using the PBPK models.

## Introduction

Radioimmunotherapy (RIT) is a cancer treatment method were radiolabeled antibodies are used to selectively irradiate tumor cells. Thus, the dose is delivered predominantly to the target while the burden to organs at risk remains acceptable [1].

^90^Y-labeled anti-CD66 antibodies are used in conditioning before blood stem cell transplantation of acute (myeloid and lymphoblastic) leukemia patients [1–4]. The mean range of the ^90^Y beta particles of 3.6 mm allows systematic and selective irradiation of leukemic cells from normal granulocytes which express CD66 on the cell surface. To ablate the marrow without disrupting the stroma, the targeted total red marrow dose is 35 Gy ([5]). The prescribed absorbed doses for red marrow from RIT are 23 Gy or 35 Gy depending on additional total body irradiation (TBI) with a prescribed absorbed dose of 12 Gy [4]. The absorbed dose to the liver was constrained to be lower than 12 Gy (TBI) or 20 Gy (no TBI), respectively. Treatment planning, i.e. the determination of the activity to administer, is performed individually as the biokinetics for red marrow and the organs at risk (kidneys, liver) differ considerably between patients. After injection of ^111^In-labeled anti-CD66 antibodies, a series of pre-therapeutic measurements are used to obtain the time-activity curves of the total body, red marrow, liver, spleen and serum. Before the introduction of physiologically based models, a sum of exponential functions was fitted to the measured pre-therapeutic biokinetic data. Subsequently, time-integrated activity coefficients were determined by (analytical) integration of the fit functions. These coefficients represent the input quantities for commonly applied nuclear medicine dosimetry software (e.g. OLINDA/EXM (Vanderbilt University, Tennessee, USA)) for the estimation of absorbed doses to the target and the organs at risk. In this dosimetric process equal pre-therapeutic and therapeutic biodistributions of the administered antibodies are assumed.

However, serum measurements during therapy in a small patient group showed that the assumption of equal biodistributions is not justified. Consequently, to be able to predict therapeutic biodistributions based on the pre-therapeutic measurements, a physiologically based pharmacokinetic (PBPK) model describing the biodistribution of radiolabeled CD66 antibodies was recently developed [3]. On the basis of the biokinetic data of eight patients, it was found that the administered number of anti-CD66 antibodies is in the same order of magnitude as the number of CD66 antigens in the patients. Thus, saturation effects occur especially for therapy, which requires higher antibody amounts due to the needed higher activity [6]. Consequently, the pre-therapeutic and therapeutic serum time-activity curves (and hence that of red marrow, liver and spleen) were considerably different. This recently developed PBPK model (based on eight patient data sets) [3] was capable of individually predicting the therapeutic biodistributions taking into account the individual pre-therapeutic measurements and the actual amounts of administered antibody in therapy. However, we showed in a following study [6] using a simple compartmental model and pre-therapeutic and therapeutic serum data of 10 patients that the immunoreactivity *r*_*im*_, which measures the preserved ability of antibodies to bind to their specific antigen after the radiolabeling procedure [7], is not close to 1. Therefore, the formerly used assumption of *r*_*im*_ *=* 1 needs to be revised.

The aims of this work were therefore to demonstrate using a larger patient group 1) the need to individually perform dosimetry and subsequent treatment planning in RIT with ^90^Y-labeled anti-CD66 antibodies, 2) that pre-therapeutic and therapeutic biodistributions in the same patient vary due to the different amounts of administered antibodies leading to a different degree of CD66 binding site saturation, and most importantly 3) that this difference in biodistributions can be accurately predicted. Therefore, we developed improved PBPK models considering the immunoreactivity of antibodies and constraints for the estimation of the red marrow antigen number. The models were tested on the available larger patient group and the model-based predictions of the biokinetics during therapy were compared to the actual measurements to demonstrate improved prediction accuracy.

## Material and Methods

### 2.1 Patients, Radiolabeling and Measurements of Anti-CD66 Monoclonal Antibody

All patients were treated in two study protocols approved by the Ethics Committee of Ulm University, and all patients gave their written informed consent. Additionally, the Ethics Committee of Ulm University approved the use of the obtained patient data for this study.

In total 27 patients with acute leukemia were investigated (21 with acute myeloid leukemia and 6 with acute lymphoblastic leukemia).

Radiolabeling of anti-CD66 antibody (BW250/183) was performed as described elsewhere [3, 8]. In short, the radionuclides (^111^In or ^90^Y) were labeled using DTPA as bifunctional chelator.

For pre-therapeutic imaging and blood sampling, (0.5 ± 0.1) mg (1 mg ≙ 6.7 nmol antibody) of radiolabeled anti-CD66 antibody with a mean ^111^In activity of (130 ± 16) MBq were administered. For therapy, (1.3 ± 0.5) mg with a mean ^90^Y activity of (3.2 ± 0.9) GBq were administered. Pre-therapeutic gamma camera imaging was performed at 2 h, 4 h, 1 d, 2 d, 3 d, 6 d post injection using a double-head gamma camera (ECAM, Siemens, Erlangen, Germany) [3]. The relative activities for the corresponding time points and organs were calculated according to MIRD pamphlet 16 [9]. Blood samples were collected at 5 min, 30 min, 1 h, 2 h, 4 h, 1 d, 2 d, 3 d and 6 d.

For quality control during therapy, blood samples were collected at 5 min, 30 min, 1 h, 2 h, 4 h, 1 d and 2 d for nine patients (P2, 6, 8–10, 13–16); at 5 min, 30 min, 1 h or 2 h, 4 h and 1 d or 2 d for five patients (P1, 3–5, 7); at 5 min, 15 min, 30 min, 1 h, 2 h, 4 h, 1 d and 2 d for two patients (P11, 12) and at 5 min, 1 h, 1 d and 2 d for 11 patients (P17-27).

### 2.2 PBPK Models

#### Global PBPK model

**Fig 1:**
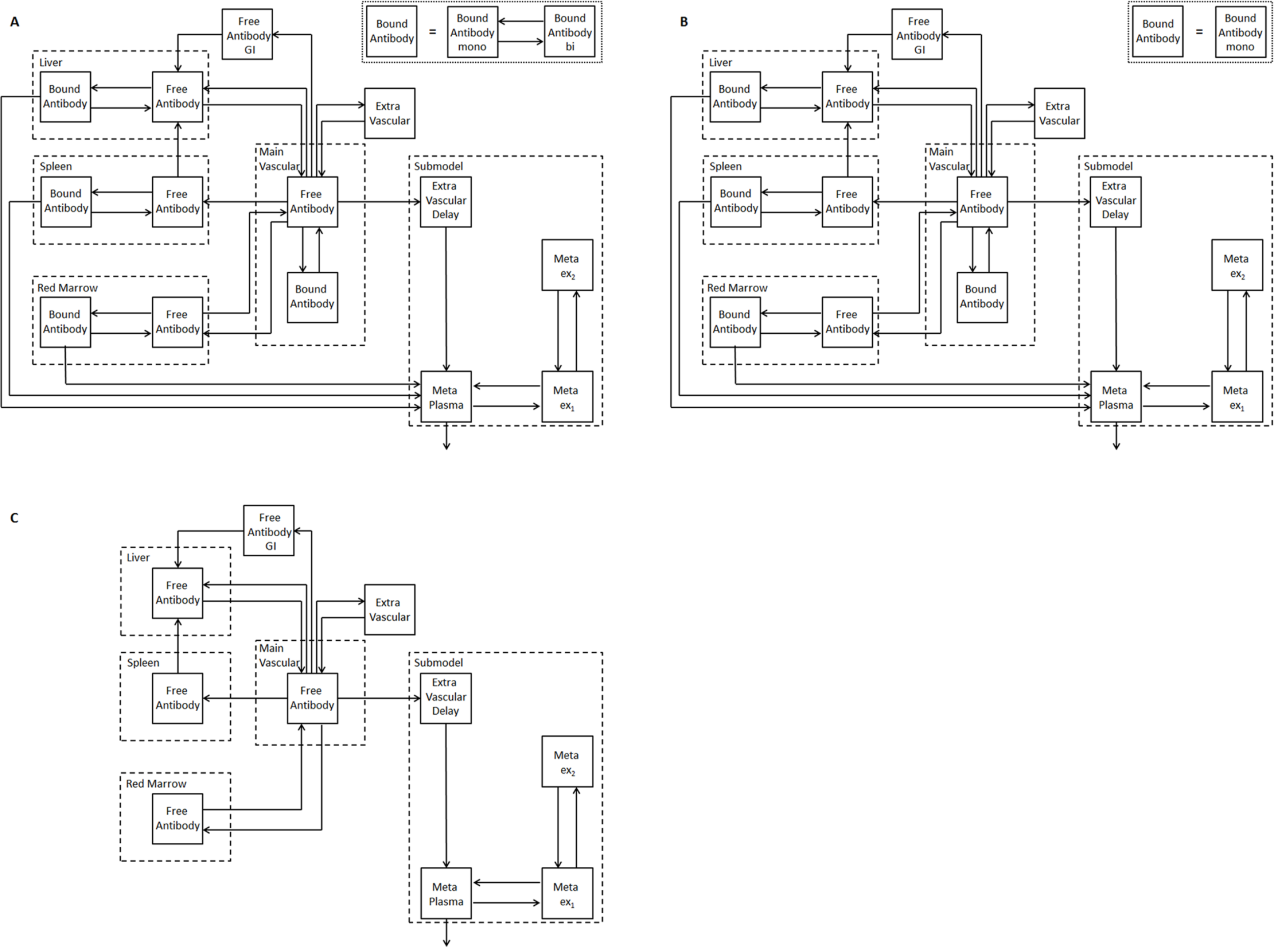
PBPK model for radiolabeled anti-CD66 monoclonal antibodies. Models for (A) fully intact (both antigen-binding sites are active, i.e. bivalent binding of antibody possible), (B) half (one antigen-binding site is active, i.e. monovalent binding) and (C) non-immunoreactive antibody (both antigen-binding sites are inactive, i.e. no binding). Due to the equivalence of both valences of the antibody, the fractions of antibodies in (A), (B) and (C) are determined as follows: With the probability r_im_ of one antibody valence being immunoreactive, the fractions of fully, half or non-immunoreactive antibody injected in (A), (B) and (C) are $r_{im}^{2},\;2r_{im}\left(1-r_{im}\right)$, and (1 − *r*_*im*_)^2^, respectively. The model consists of two equal subsystems describing the biodistribution of the labeled and unlabeled antibodies (this is true for A, B and C). The labeled and unlabeled species are competing for binding to free antigens (only A and B). The subsystems are additionally connected via physical decay, i.e. when the radiolabel decays the molecule enters the corresponding unlabeled compartment. The corresponding model equations are provided in supplement S1 Text. Radiolabeled and unlabeled antibodies are intravenously injected (main vascular compartment). The antibodies are distributed via blood flow to the main CD66 antigen expression sites. The discontinuous capillary structure of the liver, spleen and the red marrow allows the modeling of the vascular and interstitial space as one compartment. The degradation rate of bound antibody is assumed to be the same in all organs. The submodel for degraded antibody is adopted from Houston et al. [3, 28]. GI = gastrointestinal tract; Meta = metabolites in plasma; ex_1_, ex_2_ = extravascular metabolites; mono = monovalent and bi = bivalent binding.

To investigate the biodistribution of the radiolabeled anti-CD66 antibodies, the basic structure of a recently developed PBPK model was used [3]. All major biological mechanisms, and physiological properties included in the model are described by model parameters (Fig 1). A complete overview of the implemented equations, parameters and model assumptions is given in the supplement (S1 Text). In brief, after injection into the main vascular compartment the antibody is transported to the organs via blood flow. Four major antigen expressing sites, i.e. red marrow, liver, spleen and blood, were considered [3]. Non-linear saturable mono- and bivalent binding of the anti-CD66 antibody to antigen sites was explicitly modeled according to Kaufman et al. [10]. Radioactive decay and degradation of bound and unbound antibody were implemented [3].

#### Refinements to the previously published PBPK model

For the published PBPK model [3] we assumed perfect immunoreactivity *r*_*im*_ = 1. In subsequent work, however, we estimated an average immunoreactivity of *r*_*im*_ = 0.83 by simultaneously fitting a simple compartmental model to pre-therapeutic and therapeutic serum activity data [6].

This indicated that the assumption of *r*_*im*_ = 1, which was used originally [3] is not optimal. Furthermore, preliminary fits using the data of this larger patient group assuming *r*_*im*_ = 1 confirmed that with this assumption the data cannot be fitted adequately (data not shown).

Therefore, half- and non-immunoreactive antibody species were additionally considered in the PBPK model (Fig 1). To describe the biodistribution of half and non-immunoreactive labeled and unlabeled antibodies, compartmental models (part B and C in Fig 1) were added with different binding properties, however equal parameters otherwise. The distinction between fully (both antibody arms immunoreactive) and half immunoreactive (only one antibody arm immunoreactive) antibody is important as bivalent binding is considerably stronger. To account for the different but related fractions of labeled and unlabeled fully, half or non-immunoreactive antibody the fraction (probability) of antibody, the probability of an antibody arm to be immunoreactive *r*_*im*_ was introduced. This probability is estimated by fitting the entire PBPK model, which comprises all 6 circulation systems (fully, half and non-immunoreactive, labeled and unlabeled antibodies) to the pharmacokinetic data of each patient. All subunits of the model are connected by the competition for free antigens, the physical decay and probability r_im_. A detailed description including the model equation is given in the supplement S1 Text.

The association and dissociation rates *k*_*on*_ *and k*_*off*_ were fixed to typical values (*k*_*on*_ = 0.006 l/nmol/min, *k*_*off*_ = 0.06/min) [11], as fits and sensitivity analyses indicated that the exact knowledge of these parameters does not affect the biodistribution considerably. Large differences in liver and spleen biokinetics suggested an individual estimation of the fractions for unspecific uptake for liver and spleen *ex*_*l*_ and *ex*_*s*_ (“extra vascular delay” compartment, Fig 1) instead of using the values from Eger et al. [12], which were determined for a different murine antibody.

#### Different assumptions for the number of antigens in the red marrow

To further improve the PBPK model additional *a priori* knowledge was included. Two different constraints for the number of antigens in the red marrow *Ag*_*RM*_, blood *Ag*_*B*_, spleen *Ag*_*S*_ and liver *Ag*_L_ based on corresponding relations in healthy subjects [13, 14] were investigated:

$$\text{Model}\;1:\;Ag_{RM}=38\cdot Ag_{B}$$ (1)

$$Ag_{B}=\left(Ag_{L}+Ag_{S}\right)/0.9$$ (2)

$$\text{Model}\;2:\;Ag_{RM}=38\cdot Ag_{B}$$ (3)

$$Ag_{B}=\left(Ag_{L}\cdot V_{MRI,L}/V_{calc,L}+Ag_{S}\cdot V_{MRI,S}/V_{calc,S}\right)/0.9$$ (4)

*V*_*MRI*_ is the individually measured organ volume (using magnetic resonance imaging (MRI)) and *V*_*Cal*_ the calculated average organ volume according to Harris et al. and Johnson et al. [15, 16]. As the ratios in Eqs 1 and 2 are based on healthy subjects and an enlarged liver or spleen is common in acute leukemia, the weighting *V*_*MRI/*_ */V*_*cal*_ in model 2 was introduced for compensation (additional information on how these equation were derived are presented in the supplement S1 Text). For the derivation of these constraints, we assumed equal CD66 expression on all granulocytic cell forms and no considerable alteration due to acute leukemia.

### 2.3 Data Fitting and Simulation

For modeling, fitting and simulation SAAM2 (Simulation, Analysis and Modeling) software (version 2.2, The Epsilon Group, Washington, USA) was employed [17]. The computational settings were chosen as described elsewhere [3].

#### Fitting with pre-therapeutic data only and prediction of therapeutic serum curve

For parameter estimation both models were individually fitted to pre-therapeutic data of each patient. Initial values (Table 1) were taken from the literature [3, 6, 12]. Adjustable parameters (Table 1) were the number of antigens in the liver and spleen, Ag_L_ and Ag_S_, the relative blood flow to the red marrow f_RM_, the individual correction factor c_RM_ for the red marrow scaling [18], the immunoreactivity, the degradation rate of bound antibody **λ**_**db**_, the fractions for unspecific uptake for liver and spleen *ex*_*l*_ and *ex*_*s*_ and the total serum volume V. All model parameters are listed in Tables A and B in supplement S1 Text. Note that the number of antigens in the red marrow is calculated from the antigens in liver and spleen (Eqs (1–4)).

**Table 1:** Initial and estimated parameter values for all patients.

Parameter	InitialValue	Low Limit	High Limit	Fitted values mean ± SD	
				Model 1	Model 2
**Ag**_**RM**_ **[nmol]** ^†^ ^¬^	-	-	-	21±14	17±13
**Ag**_**B**_ **[nmol]** ^†^ ^¬^	-	-	-	0.58±0.39	0.50±0.38
**Ag**_**L**_ **[nmol]** ^†^	0.15	0.001	2	0.31±0.26	0.33±0.27
**Ag**_**S**_ **[nmol]** ^†^	0.15	0.001	2	0.22±0.19	0.25±0.30
**ex**_**l**_ **[unity]** ^‡^	0.19	0	1	0.235±0.089	0.226±0.091
**ex**_**s**_ **[unity]** ^‡^	0.04	0	1	0.107±0.048	0.098±0.038
**f**_**RM**_ **[%]** ^#^	3	0.01	10	0.67±0.21	0.73±0.24
c_RM_ **[unity]** ^||^	1.0	0.5	3	1.22±0.33	1.20±0.34
**λ**_**db**_ **[1/min 10** ^**–5**^ **]** §	7	1	10	6.8±1.7	6.8±1.7
**r**_**im**_ **[unity]** ^**^	0.9	0.5	Individual	0.801±0.090	0.806±0.098
**V**_**serum**_ **[l]** ^††^	Individual	1.0	10.0	2.99±0.62	3.00±0.63

All additional model parameters are fixed and their values are presented in S1 Text.

^†^ Ag_B,_ Ag_RM,_ Ag_L_ and Ag_S_ = amount of CD66 antigens in the blood, red marrow, liver and spleen, respectively.

^¬^ = Ag_RM_ and Ag_B_ are calculated according to Eqs (1–4) based on Ag_L_ and Ag_S_.

^‡^ Fractions of unspecific (extra vascular delay compartment) uptake for liver ex_l_ and spleen ex_s._

^#^ f_RM_ = relative blood flow to the red marrow.

^||^ c_RM_ = individual correction of the mean scaling factor [9] from drawn region of interest over Lumbar spine (L2-L4) to total red marrow activity.

^§^ λ_db_ = degradation rate of bound antibody.

^**^ r_im_ = immunoreactivity with lower and higher limits [29].

^††^ V_serum_ = total serum volume (Bayesian term with standard deviation as described in the methods section).

For the individual total serum volume *V*, a Bayesian term (mean *± SD)* was included:

$$V_{m,1}\pm \left(V_{m,1}-V_{BSA}\right)$$ (5)

The volume *V*_*m*,*1*_ was individually calculated using the first serum activity concentration measurement and the approximation that at this time (5 min p.i.) 100% of the activity labeled to anti-CD66 antibody is still in serum, i.e. the volume is the ratio of injected activity and measured serum sample activity concentration. The standard deviation for the Bayes term is assumed to be (*V*_*m*,*1*_ *—V*_*BSA*_), with *V*_*BSA*_ being the serum volume calculated separately for each patient by multiplication of the body surface by a factor of 2.8 for males and 2.4 for females and (1-hematocrit) [19].

For multi model inference (here two models) the Akaike information criterion was applied. The Akaike weights, which represent the probabilities for the models being most supported by the data, were calculated from fitting results of each model and data set [20].

Using the estimated parameters, the time-integrated activity coefficients for the therapeutic serum time activity curves were predicted (ã_Prediction_) for both models by integration for 20000 min (about 1% residual injected activity) starting with the time of the therapeutic injection. The coefficients were patient individually determined but model-averaged using the Akaike weights.

#### Fitting with pre-therapeutic and therapeutic data

To determine the time-integrated activity coefficients of the measured therapeutic serum data ã_Therapy_, model 2 was fitted simultaneously to the pre-therapeutic and therapeutic data of each patient. Subsequently, the fitted therapeutic serum curves were integrated yielding the serum time-integrated activity coefficients (ã_Therapy_).

#### Prediction accuracy of therapeutic serum time-activity curves

To determine the prediction accuracy, the relative deviation RD of the time-integrated activity coefficients for the predicted and measured therapeutic serum curves were calculated as follows

$$RD=\left({ã}_{Prediction}-{ã}_{Therapy}\right)\;/\;{ã}_{Therapy}.$$ (6)

#### Calculation of time-integrated activity coefficients for all other organs

For each patient and both models (with the individually fitted parameters using the pre-therapeutic data only), the pre-therapeutic and therapeutic time-integrated activity coefficients ã for the red marrow, liver, spleen, and total body were estimated. The coefficients and standard errors were calculated by numerical integration from the injection time to 20,000 min and model-averaged using the Akaike weights.

## Results

### Goodness of model fits

**Fig 2:**
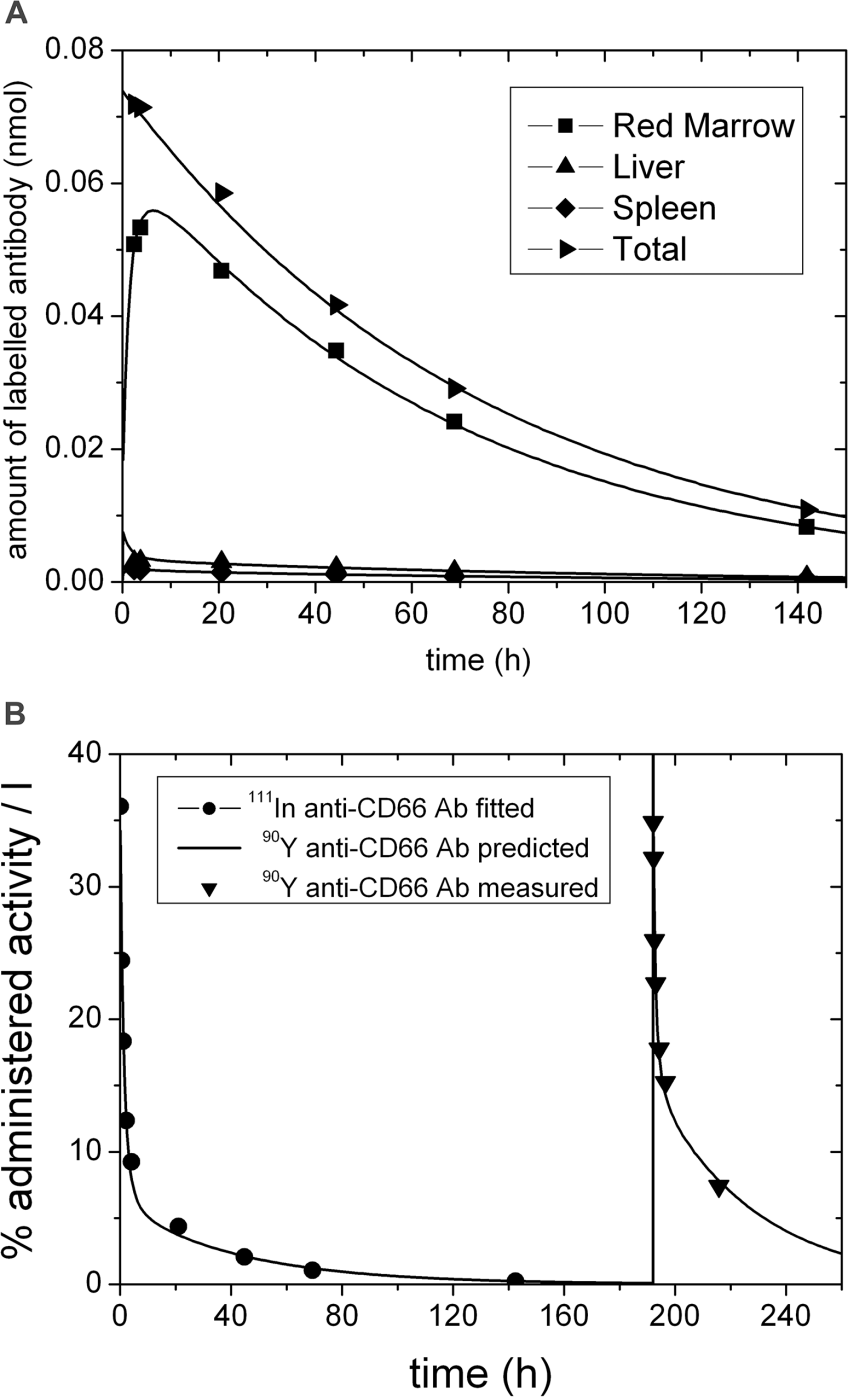
Typical biokinetic data, fit and prediction. Biokinetic data and the pertaining fitted curves using model 2 (solid lines) for labeled anti-CD66 antibodies (A) in red marrow, liver, spleen and whole body and (B) in serum. The solid line for times larger than 190 h post injection depicts the excellent prediction for the therapeutic time-activity curve based on the fitted parameters of model 2 using pre-therapeutic data only. Note that for this patient no 48 h measurement was obtained. The corresponding red marrow kinetics for all patients are presented in supplement S2 Fig.

Visual inspection showed good fits (Fig 2) for the investigated organs in all patients and for both models, except for the red marrow curve of one patient fitted with model 2. All elements of the correlation matrix were smaller than 0.84 [21] for all fits except for two patients (*Ag*_*S*_ correlation with *Ag*_*L*_) The coefficient of variation, i.e. the ratio of the standard deviation and the parameter value, of all parameters were smaller than 50% [21], except for five patients (*Ag*_*s*_, *ex*_*l*_, ex_s_ *)*. The average Akaike weights were *w*_*i*_ = 0.51 ± 0.17 and *w*_*i*_ = 0.49 ± 0.17 for model 1 and 2, respectively.

### Differences between patients

The parameters estimated by both models are presented in Table 1 showing a considerable variability (indicated by the standard deviations) between patients.

This translates into variability of the corresponding time-integrated activity coefficients (Table 2) indicating the need for patient-specific dosimetry (S1 Table). The estimated red marrow antigen numbers AgRM for models 1 and 2 are (21 ± 14) nmol and (17 ± 13) nmol, respectively. These values are of the same magnitude as the administered amounts of antibodies for therapy (((9 ± 3) nmol); supplement S1 Table) and thus lead in some patients to saturation effects.

**Table 2:** Estimated time-integrated activity coefficients ã (mean ± SD) [h] for all patients.

	ã_Pre-therapy_ ^†^	ã_Prediction_ ^*^	ã_Therapy_ ^‡^	ã_Pre-therapy_/ ã_Therapy_	ã_Prediction_/ ã_Therapy_
**Red marrow**	42.2 ± 7.7	36.5 ± 7.4	37.3 ± 7.5	1.15 ± 0.23	0.98 ± 0.28
**Liver**	6.6 ± 2.1	7.1 ± 1.9	7.0 ± 2.0	0.97 ± 0.25	1.02 ± 0.40
**Spleen**	3.1 ± 1.3	3.0 ± 0.9	2.9 ± 0.9	1.04 ± 0.27	1.02 ± 0.44
**Serum**	3.8 ± 1.4	5.0 ± 1.7	4.9 ± 1.8	0.76 ± 0.15	0.97 ± 0.20
**Whole body**	72.4 ± 3.4	74.3 ± 3.8	74.2 ± 3.8	0.98 ± 0.02	1.00 ± 0.07

† Pre-therapy = calculated from fits to the pre-therapeutic measurements

* Prediction = predicted values for the therapeutic biodistribution based on fits of the PBPK models to pre-therapeutic measurements

‡ Therapy = calculated for the therapeutic biodistribution based on fitting the PBPK models to pre-therapeutic and therapeutic measurements

Furthermore, we could confirm that the probability of antibodies to bind is on average lower than 1 (model 1: r_im_ = 0.801 ± 0.090; model 2: r_im_ = 0.806 ± 0.098) demonstrating a considerably reduced immunoreactivity. This and the considerable high standard deviation indicate the need to explicitly describe immunoreactivity in the PBPK model.

### Differences between pre-therapeutic and therapeutic biodistributions

**Fig 3:**
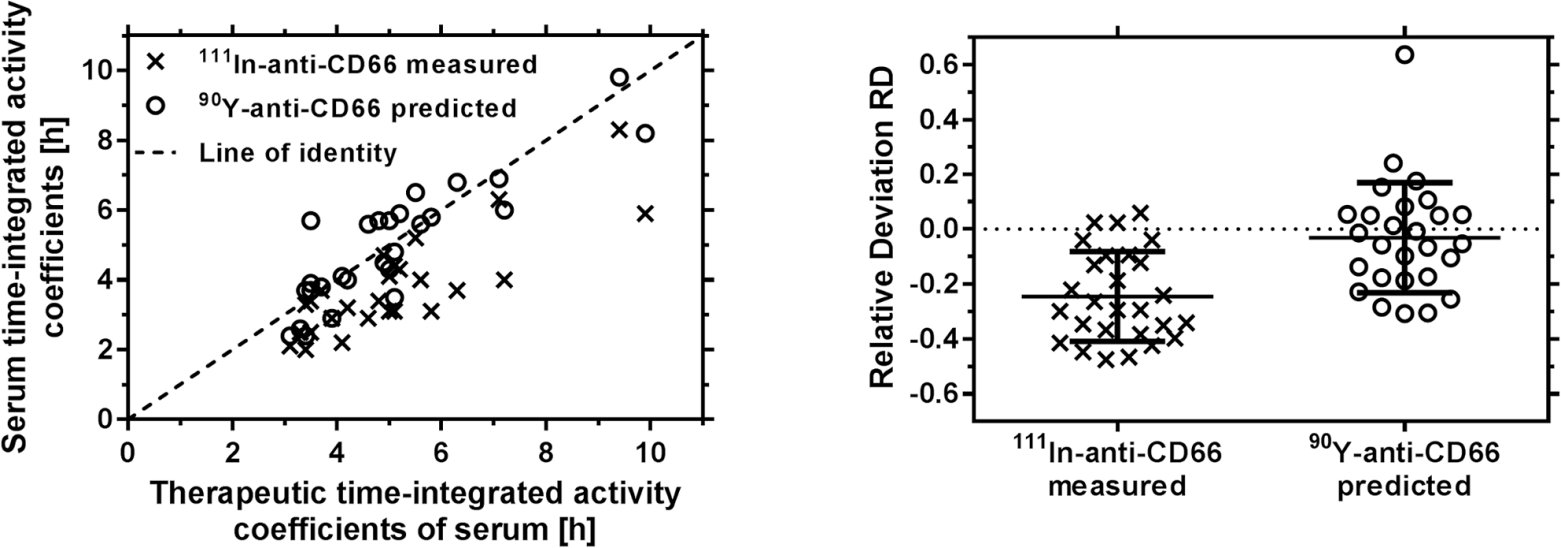
Serum time-integrated activity coefficients. (Left) Measured pre-therapeutic (^111^In) and predicted therapeutic (^90^Y) serum measurements versus actual therapeutic time-integrated activity coefficients of all patients. The application of the PBPK model allows for the prediction of therapeutic serum time-activity curves and removes the systematic offset. (Right) Relative deviation of serum time-integrated activity coefficients for all patients (scatterplots with mean and standard deviations).

The differences in pre-therapeutic or predicted and therapeutic time-integrated activity coefficients of serum curves are shown in Fig 3. The prediction accuracy of the therapeutic time-integrated activity coefficients *ã*_*therapy predicted*_ was (-25 ± 16) % assuming equal pre-therapeutic and therapeutic biodistributions (Fig 3 (right, A)). The time-integrated activity coefficients for the main antigen expressing sites are presented in Table 2. The red marrow therapeutic time-integrated activity coefficients are on average 1.2 ± 0.2 fold lower, as in some patients the number of CD66 antigens is low and the administered antibody amounts for therapy are considerably higher than for the pre-therapeutic measurements. Especially in patients with a low number of binding sites compared to the administered number of antibodies, the therapeutic time-integrated activity coefficient for serum is considerably higher during therapy (S1 Table). The differences in liver and spleen are small as increased unspecific uptake (extra vascular delay compartment) during therapy compensates for reduced CD66 specific binding.

### Prediction accuracy of therapeutic biodistributions

For the refined PBPK models the averaged mean relative deviation of the time-integrated activity coefficients for the predicted and measured therapeutic serum curves was (-3 ± 20) % (Fig 3 (right, B)). To compare this prediction accuracy with the precision of the estimated time-integrated activity coefficients, the relative standard errors (ratio of standard error and the estimate) were determined. The mean relative standard errors for total body, red marrow, spleen, liver and serum were (1.1 ± 0.4)%, (7 ± 4)%, (13 ± 4)%, (8 ± 4)% and (8 ± 4)%, respectively.

## Discussion

Radioimmunotherapy with anti-CD66 antibody is used for intensification of conditioning before stem cell transplantation in the treatment of acute leukemia. For treatment planning, the absorbed dose coefficients of the target tissue and critical organs are determined based on the individual time-integrated activity coefficients. These time-integrated activity coefficients need to be accurately determined based on pre-therapeutic measurements and pharmacokinetic modeling. In the past, it was assumed that the biokinetics of ^111^In-labeled anti-CD66 antibody is equal to the therapeutic kinetics using ^90^Y-labeled antibodies. Estimation of time-integrated activity coefficients was performed by fitting a sum of exponential functions (usually two exponential functions [21]) to the measurements (Table 3). However, we found by measuring the serum activity during therapy that the assumption of equal pre-therapeutic and therapeutic biokinetics is not justified.

**Table 3:** Overview of approaches of different complexity.

Kinetic Model	Assumptions	Corrections performed
Sum of exponential functions	Ag_RM_ ^†^ >> Amount Ab^#^	none
	RM^‡^ scaling is correct	
	r^||^_im_, _pre-therapeutic =_ r_im_, _therapeutic_	
PBPK	Ag_RM_ >> Amount Ab	for inadequate RM scaling
	r_im_, _pre-therapeutic =_ r_im_, _therapeutic_	
PBPK^§^	Ag_RM_ ~ Amount Ab	for inadequate RM scaling
	r_im_, _pre-therapeutic =_ r_im_, _therapeutic_	for different amount Ab
	r_im_, _pre-therapeutic_ ~1	for residual amount Ab
PBPK^**^Improved model	Ag_RM_ ~ Amount Ab	for inadequate RM scaling
		for different amount Ab
	r_im_, _pre-therapeutic =_ r_im_, _therapeutic_	for residual amount Ab
		for half and non-reactive Ab

^†^Ag_RM_ = number of antigens in the red marrow

^‡^RM = red marrow

^#^ Ab = antibody

^||^r_im_ = immunoreactivity

^§^ = recently developed PBPK model [3] based on data sets of 8 patients

^**^ = presented refined PBPK model based on data sets of 27 patients

We could show for eight patients that saturation effects cause these differences and that a PBPK model is capable to predict the occurring changes [3]. In contrast to PBPK modeling and fitting, exponential functions fitting can neither be used to correct for inaccurate scaling of the red marrow activity from the lumbar spine to total red marrow mass, nor the change in biodistributions due to using different amounts of antibody (pre-therapeutic vs therapeutic) and residual labeled (~8%) and unlabeled (~20%) antibody from the pre-therapeutic measurements.

This recently developed PBPK model [3] does not account for reduced immunoreactivity. However, an investigation using a simple compartmental model and pre-therapeutic and therapeutic serum data of 10 patients showed that the distribution of immunoreactive, half- and non-immunoreactive antibody has to be explicitly modeled to accurately determine the time-integrated activity coefficients [6].

In this work, the biokinetic data of 27 patients were used to demonstrate that patient-specific dosimetry is required in RIT with ^90^Y-labeled anti-CD66 antibody and that PBPK models are needed for accurate prediction of the therapeutic biodistributions. This original PBPK model [3] was refined by adding compartments for half intact and non-immunoreactive antibodies (Fig 1). Furthermore, two constraints for the amount of antigens in the serum and the red marrow were implemented (model 1, 2). These refined PBPK models could be adequately fitted to all data sets. Both models were used for parameter inference, i.e., time-integrated activity coefficients were weighted according to the corresponding Akaike weights [20]. The mean deviation of the averaged predicted time-integrated activity coefficients was RD = (-3 ± 20) %.

To identify the impact of considering a reduced immunoreactivity in modeling and fitting, we investigated this larger patient group in addition to the results shown in Fig 3 using the previously published model [3]. This model inherently assumes a perfect immunoreactivity, i.e. rim = 1. The obtained relative deviation of the therapeutic serum time-activity curves was RD = (16 ± 46) %. The large improvement of the new model RD = (-3 ± 20) %) with respect to mean, which is around zero, i.e. essentially no existing systematic deviation, and a lower variation demonstrates that the assumption of fully immunoreactive antibodies needs to be abandoned. This is also supported by the individually fitted immunoreactivities in the new model; on average the fitted immunoreactivity (0.8) was comparable to literature values [22], however very low (smaller than 0.7) for five patients. Measuring immunoreactivity would have strengthened the validation, but unfortunately it was only measured during development of the radiolabelling procedure and therefore not for each patient. Note that it is not clear how a measured value of immunoreactivity is related to the probability r_im_ of one antibody arm being immunoreactive used to calculate the ratios of fully, half and non-immunoreactive antibodies. However, for future investigations, immunoreactivity may be measured in vitro and compared to PBPK model results.

Here we employed an important advantage of using PBPK models, i.e. the simultaneous fit of all measured data. This allowed the use of the more accurate serum and total body measurement to gain additional information for the estimation of organ parameters. The background correction of the red marrow in planar gamma camera images is difficult and might lead to over- or under-estimation of the true value. However, more accurate whole body and serum measurements help to increase the accuracy for the estimation of red marrow parameters. Specifically, fitting all data in one objective function allowed correcting the commonly used scaling factor for the ratio of red marrow in L2-L4 of the spine to total body red marrow. Usually only the red marrow activity of the lumbar spine (region of interest including L2-L4) is measured and then scaled by the patient height [9]; the PBPK modeling allows to estimate the individual total activity of the red marrow from the measured data. The estimated scaling factor by patient height was up to two times higher than the corrected value found by the PBPK model, which clearly shows the necessity to correct the RM scaling.

The calculated red marrow time-integrated activity coefficient in an individual patient was up to a factor of 1.7 smaller for therapy than for pre-therapy. The assumption of equal biodistributions underestimated the serum time-integrated activity coefficient ã_serum_ and therefore overestimated the coefficient for the red marrow ã_RM_ in 24 of 27 patients. The results confirm that the assumption of equal pre-therapeutic and therapeutic biodistribution leads to inadequate predictions.

The red marrow antigen number Ag_RM_ is the most important parameter, which is not known *a priori*. Therefore, the red bone marrow antigen number was estimated assuming a ratio of red bone marrow (all forms) and circulating granulocytes (all forms) of 38 [13]. Although this factor was derived from healthy subjects and it is known that ALL and AML may alter the number of cells in blood and the red marrow, the relative deviation of the time-integrated activity coefficient calculated from the predicted and measured serum curve shows a negligible systematic error of -3% for the population. In one patient this ratio led to a considerable underestimation of the number of antigens in the red marrow (Fig 3B). A correlation of the ratio of spleen to body weight with the deviations of the predicted and measured therapeutic serum time-activity curve was significant (p < 0.05). The lowest ratio was found for the patient with the highest deviation indicating that the used assumption of model 2 (Eqs (3 and 4)) is underestimating the number of antigens of the red marrow for patients with a low spleen to body weight ratio. Another assumption in deriving Eqs (1–4) was an equal CD66 antigen expression for all granulocyte cell forms [23], which might not be always the case. Based on a spherical shape of the cells with a typical radius of 6 μm [24] the average cell mass is about 10^–9^ g. Thus, with an assumed total red marrow CD66-positive cell number per patient of approximately 10^12^ (corresponding to 1 kg) and the weighted mean red marrow antigen number Ag_RM_ (19 nmol), we obtain a CD66 antigen expression of approximately 10^4^/cell *in vivo*, which is in a typical range.

The obtained parameter value for the degradation rate is comparable to RIT with anti-CD45 antibody [25]. The fractions of liver and spleen for unspecific (extra vascular delay compartment) uptake show high variability and are on average higher than those reported [12]. As more than one biological mechanism is lumped together it is not entirely clear whether this might stem from different uptake due to FcRn binding or direct metabolism [26]. The fitted relative red marrow blood flow f_rm_ is four times lower than in healthy humans possibly caused by an alteration of the blood flow due to leukemia. Although antibodies can pass freely between large pores of the capillary wall of the red marrow tissue [12], modeling the vascular and interstitial space as one compartment (lumping of red marrow tissues spaces) might be an oversimplification.

We used the kinetics of the therapeutic serum time-activity curves for validation. The individual serum time-activity curve represents an important measure as it mirrors the number of unbound antibody, which depends on the number of bound antibody. The number of bound antibody is in turn determined by the red marrow antigen number. Direct measurements of the organ activities are nevertheless desirable for validation. These however are challenging for the red bone marrow based on bremsstrahlung of ^90^Y. If properly implemented, such measurements during therapy would certainly be helpful for further validation [27].

Besides the achieved improvement regarding the prediction accuracy the developed PBPK model might also be used to suggest further steps to increase the uptake in the red marrow by simulating other therapeutic scenarios. For example, for patients with a smaller number of antigens in the red marrow (here for 11 patients Ag_RM_ ≤ 10 nmol) a reduction of the used amount of antibody ((9 ± 3) nmol for therapy) would considerably increase the fraction of bound antibody. Thus, adequate PBPK models and the improvements presented here are essential to accurately describe the biodistribution of radiolabeled antibodies *in vivo* in case that saturation is relevant or the immunoreactivity deviates considerably from unity. The explicit modeling of immunoreactivity showed that the former assumption of perfect immunoreactivity was invalid (r_im_ ~ 0.8). Furthermore, these models allow accounting for residual antibodies from pre-therapeutic measurements, which may further decrease the fraction of bound antibodies radiolabeled with the therapeutic radionuclide. Clearly, higher immunoreactivity would also be beneficial.

## Conclusions

In radioimmunotherapy with ^90^Y-labeled anti-CD66 antibodies individual treatment planning is needed because of large biological variability between patients.

Differences between pre-therapeutic and therapeutic biodistributions occur because (1) the numbers of applied antibodies and available CD66 binding sites are in the same order of magnitude, (2) considerable more antibody is given for therapy than for the pre-therapeutic measurements (different saturation effects), (3) residual antibody from pre-therapeutic measurements is still present.

For the red marrow a 1.2 ± 0.2 fold lower therapeutic to pre-therapeutic time-integrated activity coefficient was estimated. In addition it was found, that the scaling from red marrow of the lumbar spine to the entire red marrow based on body height is not appropriate (on average 1.2 too large) for this population. These differences will lead to under-treatment of the red marrow if they are not considered in the estimation of the time-integrated activity coefficients

PBPK modeling allows accurate prediction of the individual time-integrated activity coefficients in radioimmunotherapy with ^90^Y-labeled anti-CD66 antibodies taking into account 1) individual biological differences such as the number of CD66 antigens in the red marrow or the scaling factor from lumbar spine to the entire red marrow and 2) the administration of different amounts of antibody and residual antibody from the pre-therapeutic measurements. Fitting a sum of exponential functions to pre-therapeutic data (assuming equal pre-therapeutic and therapeutic biodistributions) considerably overestimates the time-integrated activity coefficients.

## Supporting Information

S1 Fig Prediction Kinetics. Therapeutical serum measurements (dots) together with the predicted serum kinetics (lines) based on the pre-therapeutic measurements and the PBPK model for 27 patients.
(PDF)

S2 Fig Optimized Red Marrow Biodistribution. Red marrow kinetics measured pre-therapeutically (symbols) together with the model fits (lines) for all 27 patients showing the large interindividual variation. For better visibility, the patient group is displayed on two graphs (A: P1-13; B: P14-27).
(PDF)

S1 Table Patient Data and Fitting Results. Overview of gamma camera and serum measurements for all 27 patients; fitted and predicted therapeutic time-integrated activity coefficients and administered and estimated red marrow antigen numbers.
(XLSX)

S1 Text Model Equations and Parameters. Model equations and parameters for the description of the biodistribution of fully-, half- and non-immunoreactive labeled and unlabeled anti-CD66 antibodies.
(PDF)
