# Supplementary figures and images for: Physiologically Based Pharmacokinetic Modeling Is Essential in 90Y-Labeled Anti-CD66 Radioimmunotherapy
Source: PLoS One. 2015 May 26;10(5):e0127934. doi: 10.1371/journal.pone.0127934 (PMC4444288; doi:10.1371/journal.pone.0127934)

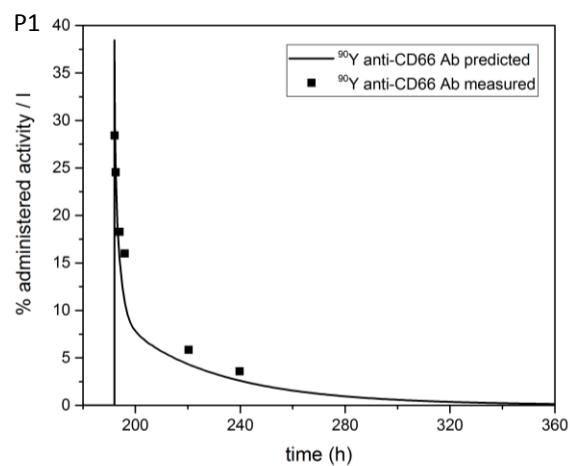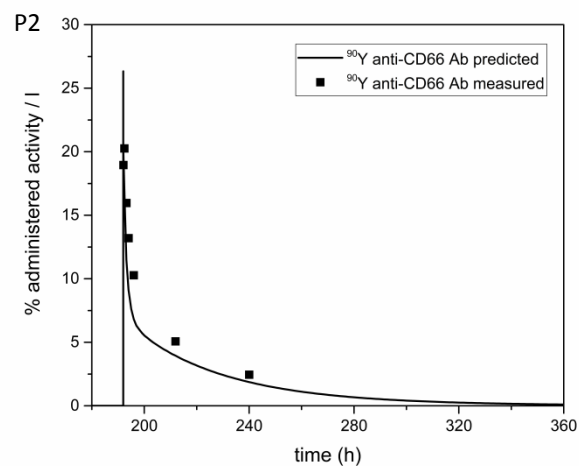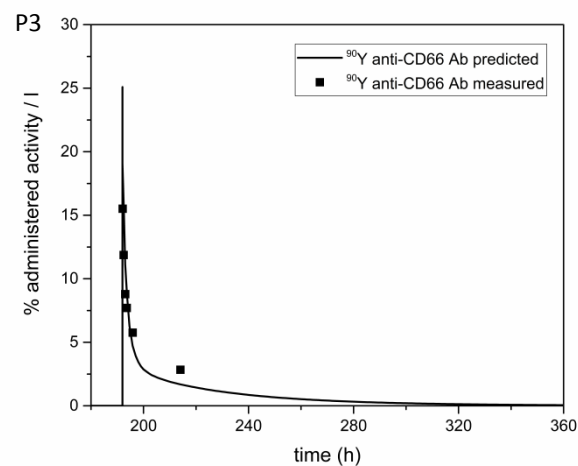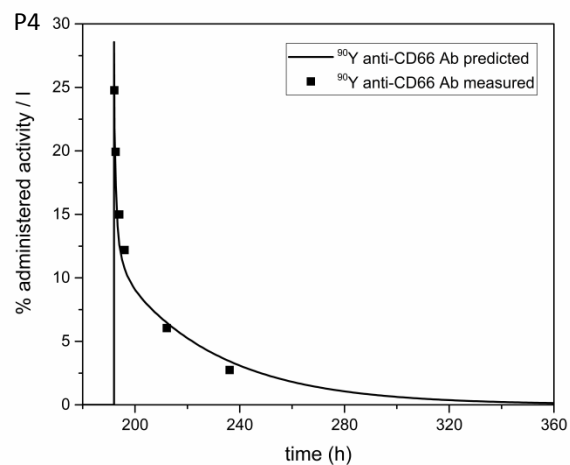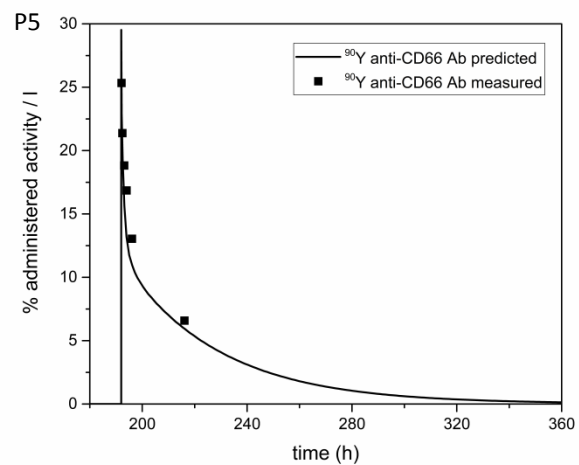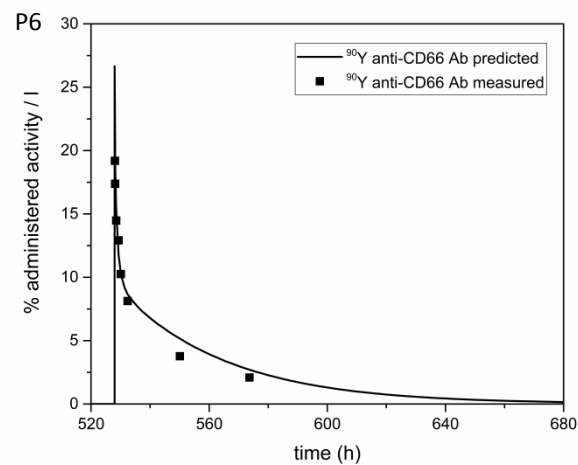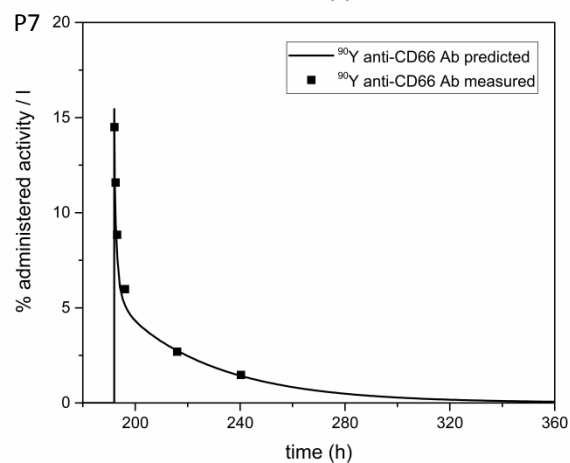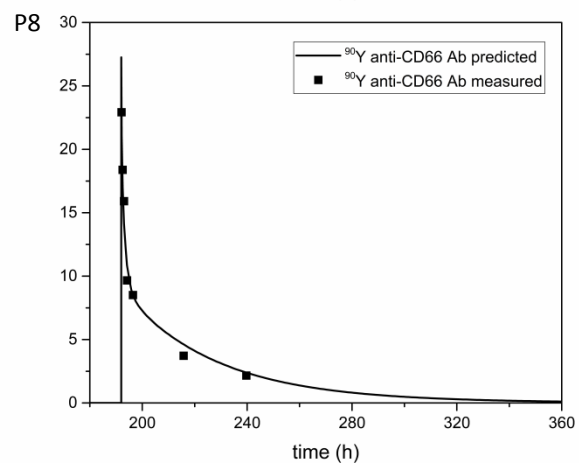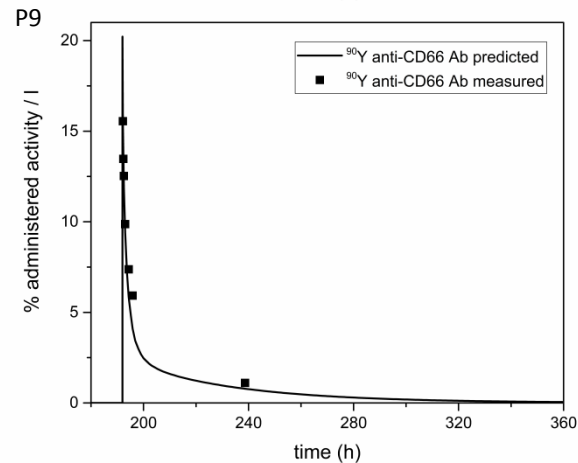

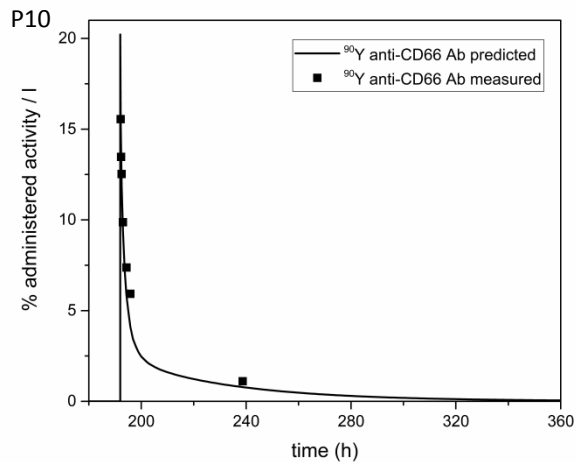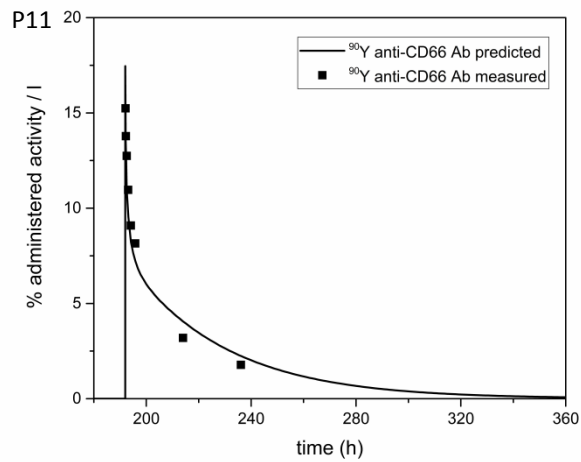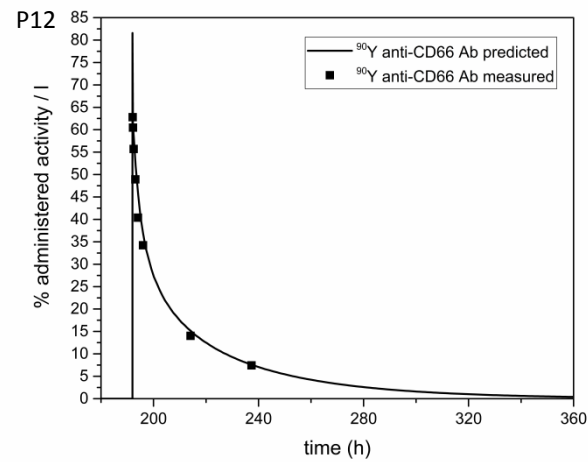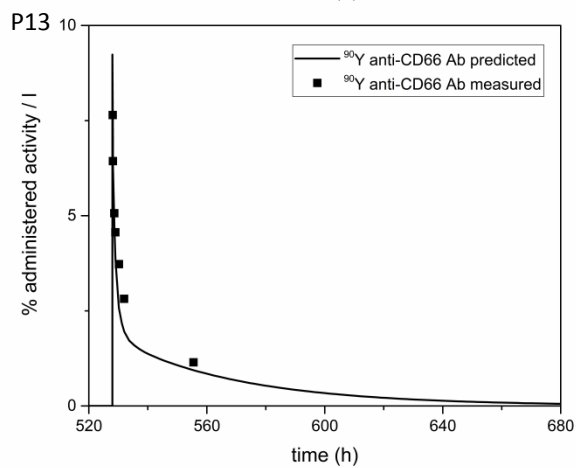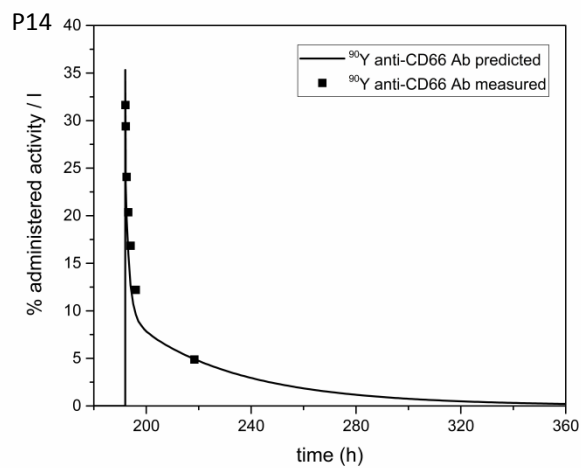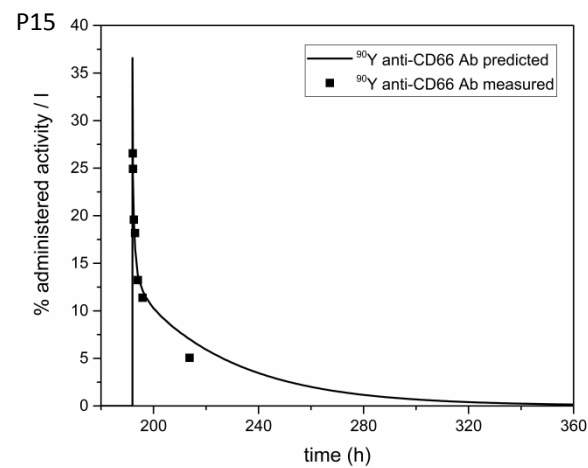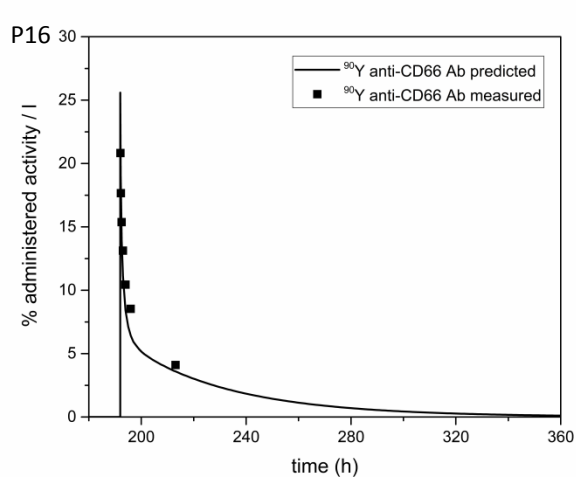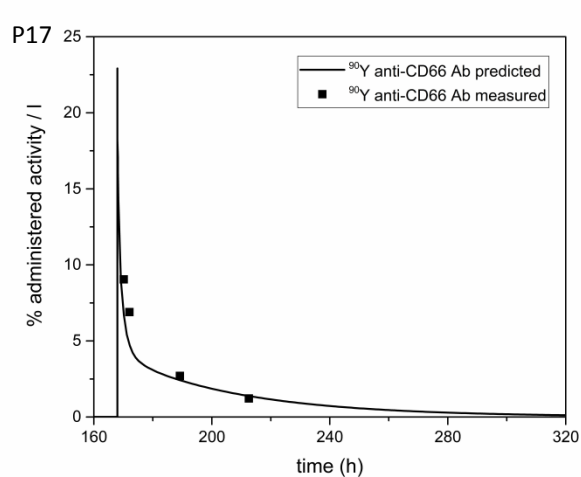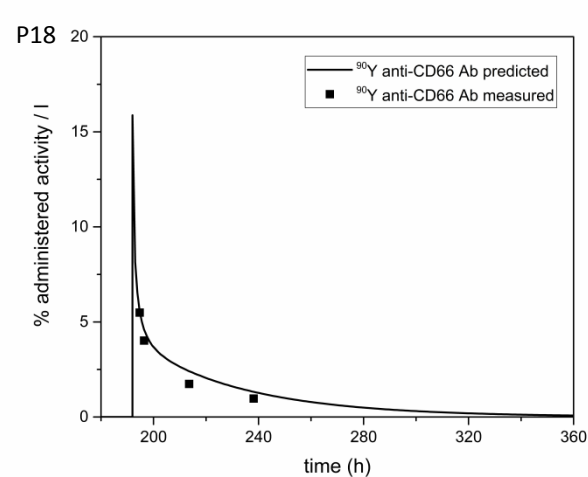

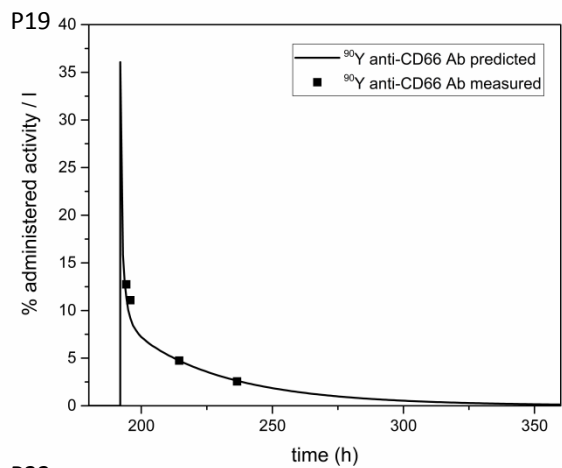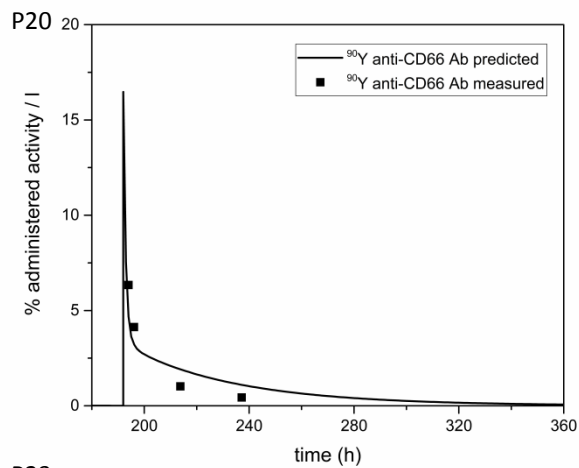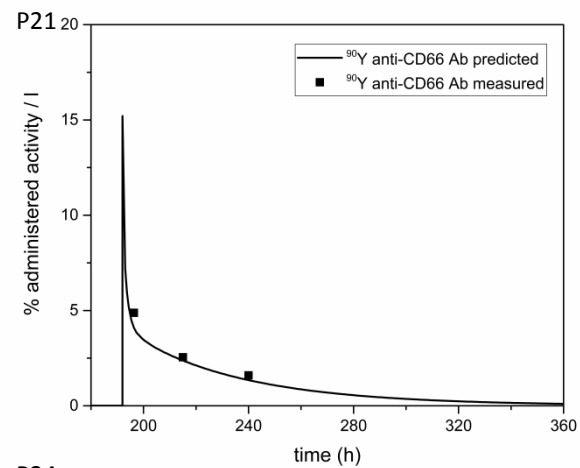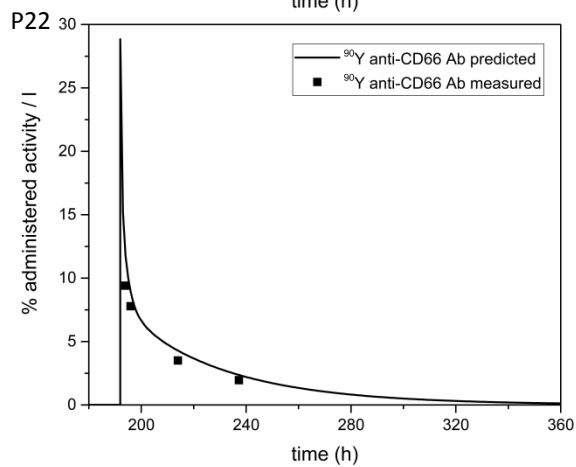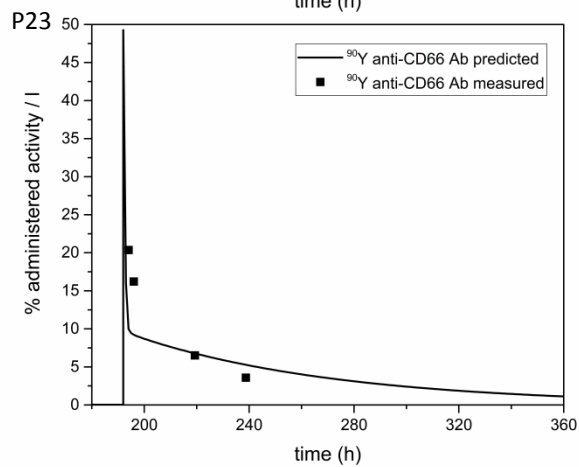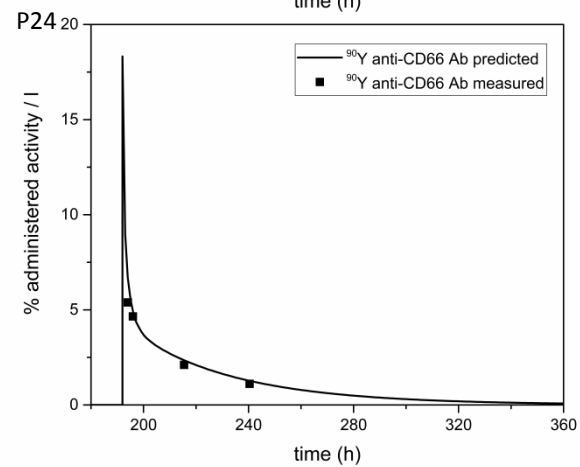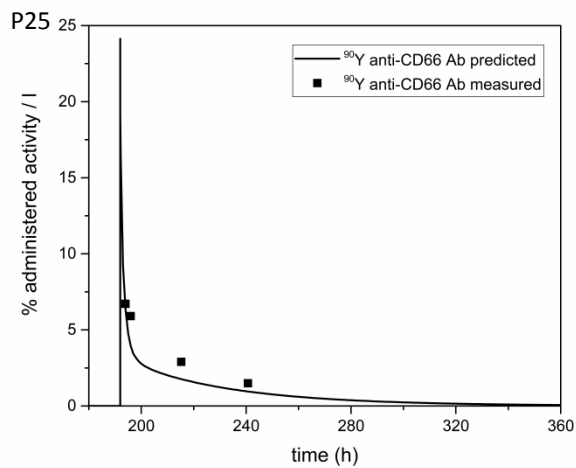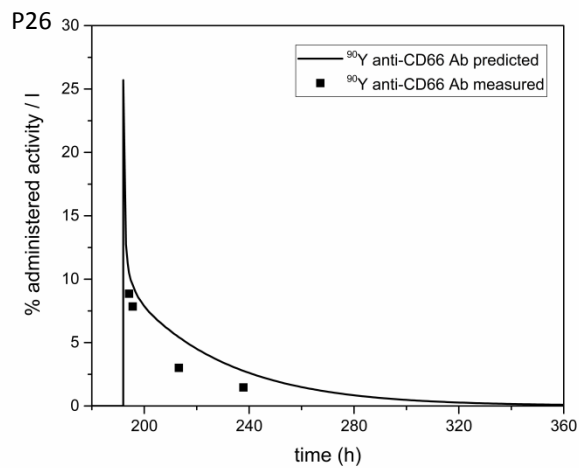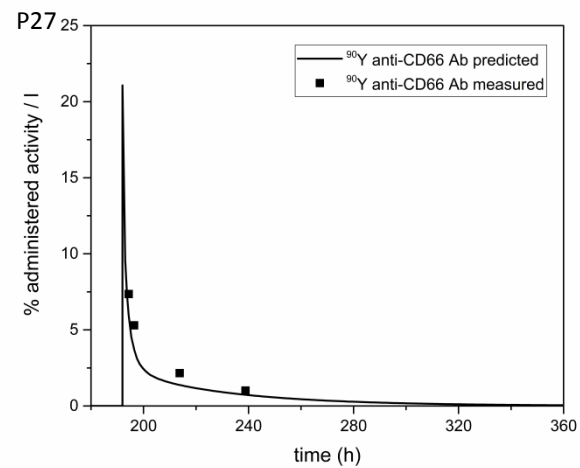

Supplement: S1 Fig — Therapeutical serum measurements (dots) together with the predicted serum kinetics (lines) based on the pre-therapeutic measurements and the PBPK model for 27 patients. (PDF) [file pone.0127934.s001.pdf]

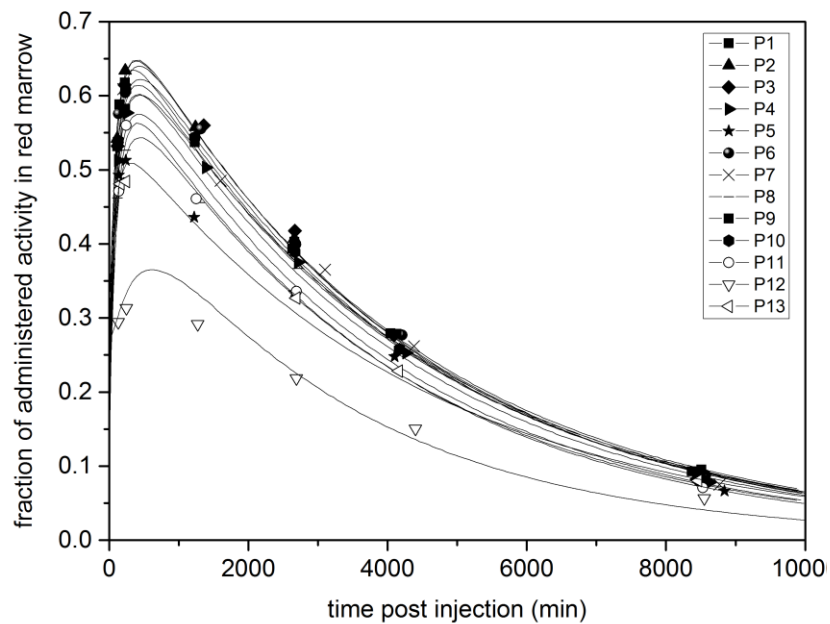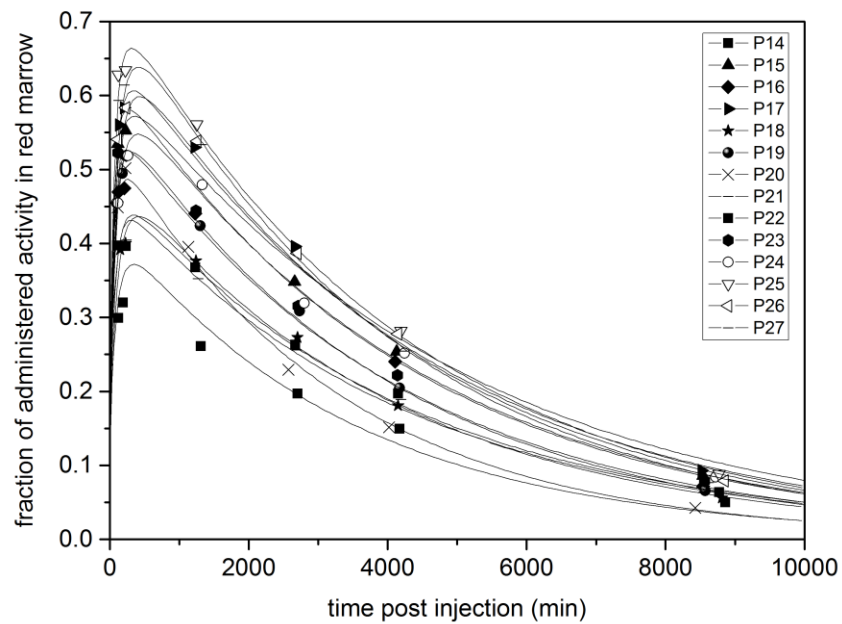

Supplement: S2 Fig — Red marrow kinetics measured pre-therapeutically (symbols) together with the model fits (lines) for all 27 patients showing the large interindividual variation. For better visibility, the patient group is displayed on two graphs (A: P1-13; B: P14-27). (PDF) [file pone.0127934.s002.pdf]
