# Supplementary material for: Physiologically Based Pharmacokinetic Modeling Is Essential in 90Y-Labeled Anti-CD66 Radioimmunotherapy
Source: PLoS One. 2015 May 26;10(5):e0127934. doi: 10.1371/journal.pone.0127934 (PMC4444288; doi:10.1371/journal.pone.0127934)
Supplement: S1 Text — Model equations and parameters for the description of the biodistribution of fully-, half- and non-immunoreactive labeled and unlabeled anti-CD66 antibodies. (PDF) [file pone.0127934.s004.pdf]

Supplement: Model equations and parameters

The following equations and parameters describe the biodistribution for fully-, half-, and non-immunoreactive labeled (indexed with *) and unlabeled anti-CD66 antibodies in patients suffering from acute leukemia. All variables are defined in Tables A and B.

Volumes of distribution

Antibody can freely pass [1] between the interstitial and vascular space of the liver, spleen and red marrow [2]. The available volume fraction for antibody is low [3].

Therefore, the antibody distribution volume is approximated as fraction of the total blood pool, using values reported by Leggett et al. [5]:

$$\begin{aligned} V_{RM} &= V \cdot 0.04 \\ V_{GI} &= V \cdot 0.076 \\ V_L &= V \cdot 0.1 \\ V_S &= V \cdot 0.014 \end{aligned} \qquad (1)$$

Constraint for antigen numbers (i = red marrow, liver, spleen, blood):

$$\begin{aligned}Ag_i = Ag_{0,\,i} - AgAb_{\mathrm{fa}\,mono,\,i} - AgAb^*_{\mathrm{fa}\,mono,\,i} - 2\cdot AgAb_{\mathrm{fa}\,bi,\,i} - 2\cdot AgAb^*_{\mathrm{fa}\,bi,\,i} \\ - AgAb_{\mathrm{ha}\,mono,\,i} - AgAb^*_{\mathrm{ha}\,mono,\,i}\end{aligned} \quad (2)$$

Bound antibody

$$k_{on,\,bi}\cdot[Ag]_s = \frac{k_{on,\,mono}\cdot \mathrm{E}\cdot Ag_i}{4\cdot\pi\cdot r_{cell}{}^2 N_{cells,\,i}} \equiv k_{on,\,mono}\cdot Ag_i\cdot\alpha_i \quad (3)$$

$[Ag]_s$ represents the surface concentration of unbound antigens and α_i is an abbreviation defined in Eq. (4) to simplify the notation of the differential equations. The ratio E = $k_{on,\ bi}$ / $k_{on,\ mono}$ used in the literature [6] basically stems from the conversion from bulk to surface concentrations using average binding site concentrations rather than a statistical mechanical model of the surface.

Assumptions for cell numbers

Equal CD66 expression on all granulocytic cell forms and no considerable alteration due to disease is assumed. The average number of CD66 expressing cells is assumed to be 38 fold higher in the red marrow than in the blood [7] (for circulating cells).

$$\begin{aligned}N_{cells,\,RM} &= 38\cdot N_{cells,\,B,c} \\ Ag_{0,\,RM} &= 38\cdot Ag_{0,\,B}\end{aligned} \quad (4)$$

The antigen numbers $Ag_{0,\,L}$ and $Ag_{0,\,S}$ for liver and spleen are assumed to be proportional to the number of marginating granulocytes $N_{cells,\,L,m}$ and $N_{cells,\,S,m}$. To establish a relationship between the antigen numbers for liver and spleen and the number of circulating granulocytes, literature values were used [8]. The numbers of

circulating and marginating $N_{cells,\ B,c}$ and $N_{cells,\ B,m}$ are 40 % and 60% of the total blood granulocytes pool, respectively.

$$N_{cells,\ B,m} = 1.5 \cdot N_{cells,\ B,c} \qquad (5)$$

According to [8], the number of marginating granulocytes in liver and spleen $N_{cells,\ L,m}$ and $N_{cells,\ S,m}$ are equal to 60% of the total blood marginating granulocytes.

$$N_{cells,\ B,m} = (N_{cells,\ L,m} + N_{cells,\ S,m}) \cdot 0.6 \qquad (6)$$

Therefore, the sum of the numbers of fix CD66 positive cells in the liver and spleen is set equal to 90% of the circulating CD66 positive cells in the blood (Model 1).

$$N_{cells,\ B,c} = (N_{cells,\ L,m} + N_{cells,\ S,m}) \cdot 0.9 \qquad (7)$$

Model 2 includes weighting factors which take into account the actual sizes of the spleen or liver, as larger deviations to the calculated size (based on parameters such as age and body weight [9,10]) might alter the average number of CD66 positive cells in these organs.

<u>Model 1:</u>

$$Ag_{0,\ B} = (Ag_{0,\ L} + Ag_{0,\ S}) \cdot 0.9 \qquad (8$$

<u>Model 2:</u>

$$Ag_{0,\ B} = (Ag_{0,\ L} \cdot \frac{V_{MRI,\ L}}{V_{Cal,\ L}} + Ag_{0,\ S} \cdot \frac{V_{MRI,\ S}}{V_{Cal,\ S}}) \cdot 0.9 \qquad (9)$$

Injections:

Total amount of radiolabeled and unlabeled injected antibody

$$
\begin{aligned}
Ab^{*}_{inj,PT} &= a \cdot f_{l,PT} \cdot Ab_{total,PT} \cdot \frac{A_{inj,PT}}{A_{total,PT}} \\
Ab_{inj,PT} &= (1 - a \cdot f_{l,PT}) \cdot Ab_{total,PT} \cdot \frac{A_{inj,PT}}{A_{total,PT}} \\
Ab^{*}_{inj,T} &= b \cdot f_{l,T} \cdot Ab_{total,T} \cdot \frac{A_{inj,T}}{A_{total,T}} \\
Ab_{inj,T} &= (1 - b \cdot f_{l,T}) \cdot Ab_{total,T} \cdot \frac{A_{inj,T}}{A_{total,T}}
\end{aligned} \tag{10}
$$

The antibody (composed of labeled and unlabeled fractions) was injected as a bolus into the "free main vascular compartments" (Figure 1) for pre-therapeutic measurements (PTM) and therapy. The individually determined radiochemical purities for therapy, *a*, and pre-therapeutic measurements, *b*, were used.

Amount of fully intact radiolabeled and unlabeled injected antibody

$$
\begin{aligned}
Ab^{*}_{fa,inj,PT} &= Ab^{*}_{inj,PT} \cdot r^2 \\
Ab^{*}_{fa,inj,T} &= Ab^{*}_{inj,T} \cdot r^2
\end{aligned} \tag{11}
$$

$$
\begin{aligned}
Ab_{fa,inj,PT} &= Ab_{inj,PT} \cdot r^2 \\
Ab_{fa,inj,T} &= Ab_{inj,T} \cdot r^2
\end{aligned} \tag{12}
$$

Amount of half immunoreactive radiolabeled and unlabeled injected antibody:

$$
\begin{aligned}
Ab^{*}_{ha,inj,PT} &= Ab^{*}_{inj,PT} \cdot 2 \cdot (1 - r) \cdot r \\
Ab^{*}_{ha,inj,T} &= Ab^{*}_{inj,T} \cdot 2 \cdot (1 - r) \cdot r
\end{aligned} \tag{13}
$$

$$
\begin{aligned}
Ab_{ha,inj,PT} &= Ab_{inj,PT} \cdot 2 \cdot (1 - r) \cdot r \\
Ab_{ha,inj,T} &= Ab_{inj,T} \cdot 2 \cdot (1 - r) \cdot r
\end{aligned} \tag{14}
$$

Amount of non-immunoreactive radiolabeled and unlabeled injected antibody:

$$\begin{aligned} Ab^{*}_{na,inj,PT} &= Ab^{*}_{inj,PT} \cdot (1-r)^{2} \\ Ab^{*}_{na,inj,T} &= Ab^{*}_{inj,T} \cdot (1-r)^{2} \end{aligned} \tag{15}$$

$$\begin{aligned} Ab_{na,inj,PT} &= Ab_{inj,PT} \cdot (1-r)^{2} \\ Ab_{na,inj,T} &= Ab_{inj,T} \cdot (1-r)^{2} \end{aligned} \tag{16}$$

Data assigned to compartments:

Liver

$$\begin{aligned} data_{L} = {} & AgAb^{*}_{fa\,\mathrm{bi},L} + AgAb^{*}_{fa\ \mathrm{mono},L} + Ab^{*}_{fa,L} + AgAb^{*}_{ha\ \mathrm{mono},L} + Ab^{*}_{ha,L} + Ab^{*}_{na,L} \\ & + ex_{l} \cdot (Ex^{*}_{fa} + Ex^{*}_{ha} + Ex^{*}_{na}) + \frac{V_{L}}{V} \cdot (Meta^{*}_{fa,P} + Meta^{*}_{ha,P} + Meta^{*}_{na,P} \\ & + AgAb^{*}_{fa\,\mathrm{bi},B} + AgAb^{*}_{fa\ \mathrm{mono},B} + AgAb^{*}_{ha\ \mathrm{mono},B}) + \mathrm{int}_{L} \cdot (Ab^{*}_{fa\,\mathrm{int}} + Ab^{*}_{ha\,\mathrm{int}} + Ab^{*}_{na\,\mathrm{int}}) \end{aligned} \tag{17}$$

Spleen

$$\begin{aligned} data_{S} = {} & AgAb^{*}_{fa\,\mathrm{bi},S} + AgAb^{*}_{fa\ \mathrm{mono},S} + Ab^{*}_{fa,S} + AgAb^{*}_{ha\ \mathrm{mono},S} + Ab^{*}_{ha,S} + Ab^{*}_{na,S} \\ & + ex_{S} \cdot (Ex^{*}_{fa} + Ex^{*}_{ha} + Ex^{*}_{na}) + \frac{V_{S}}{V} \cdot (Meta^{*}_{fa,P} + Meta^{*}_{ha,P} + Meta^{*}_{na,P} \\ & + AgAb^{*}_{fa\,\mathrm{bi},B} + AgAb^{*}_{fa\ \mathrm{mono},B} + AgAb^{*}_{ha\ \mathrm{mono},B}) + \mathrm{int}_{S} \cdot (Ab^{*}_{fa\,\mathrm{int}} + Ab^{*}_{ha\,\mathrm{int}} + Ab^{*}_{na\,\mathrm{int}}) \end{aligned} \tag{18}$$

Serum

$$data_{Serum} = \frac{(Ab^{*}_{fa\,P} + Ab^{*}_{ha\,P} + Ab^{*}_{na\,P}) + \frac{V_{P}}{V}(Meta^{*}_{fa,P} + Meta^{*}_{ha,P} + Meta^{*}_{na,P})}{V_{P}} \tag{19}$$

Red Marrow

$$data_{RM_{L2-L4}} \cdot K = c_{rm} \cdot \begin{bmatrix} AgAb^{*}_{fa\,\mathrm{bi},RM} + AgAb^{*}_{fa\ \mathrm{mono},RM} + Ab^{*}_{fa,\,RM} \\ + AgAb^{*}_{ha\ \mathrm{mono},RM} + Ab^{*}_{ha,RM} + Ab^{*}_{na,RM} \\ + \frac{V_{RM}}{V} \cdot (Meta^{*}_{fa,P} + Meta^{*}_{ha,P} + Meta^{*}_{na,P} + AgAb^{*}_{fa\,\mathrm{bi},B} \\ + AgAb^{*}_{fa\ \mathrm{mono},B} + AgAb^{*}_{ha\ \mathrm{mono},B}) + x \end{bmatrix} \tag{20}$$

x = underlying activity from arteries and veins in the red marrow. In $ROI_{L2\text{-}L4}$ these are considered using equation (18)

$$x = \frac{V_{ROI,L2-L4}}{V_P}\begin{bmatrix} (Ab^*_{fa\,P} + Ab^*_{ha\,P} + Ab^*_{na\,P}) \\ + \frac{V_P}{V}(Meta^*_{fa,P} + Meta^*_{ha,P} + Meta^*_{na,P} + AgAb^*{}_{fa\,\mathrm{bi},B} \\ + AgAb^*{}_{fa\ \mathrm{mono},B} + AgAb^*{}_{ha\ \mathrm{mono},B}) \end{bmatrix} \quad (21)$$

TABLE A. Definition of parameters for data assignment

	Description	Value	Unit	Source
ex_L	liver fraction of extravascular delay compartment	estimated	unity	[1]
ex_S	spleen fraction of extravascular delay compartment	estimated	unity	[1]
int_L	liver fraction of nonsaturable compartment	0.04	unity	[1]
int_S	spleen fraction of nonsaturable compartment	0.04	unity	[1]
K	height corrected scaling factor of reverence man implemented in UlmDos to scale from L2-L4 (lumbar spine) to the entire red marrow	170cm/ height (cm)/0.06665	unity	[11]
c_{rm}	individual correction for inadequate K	estimated	unity	

Description of global model: Part A, fully immunoreactive antibody

Bivalent compartments

$$\begin{aligned}\frac{d}{dt}\left[AgAb_{fa\,bi,i}\right]\cdot V_i &= k_{on,\,mono}\cdot\alpha_i\cdot Ag_i\cdot AgAb_{fa\,mono,i}-2\cdot k_{off}\cdot AgAb_{fa\,bi,i}\\&\quad-\lambda_{db}\cdot AgAb_{fa\,bi,i}+\lambda_{phy}\cdot AgAb^{*}_{fa\,bi,\,i}\\\frac{d}{dt}\left[AgAb^{*}_{fa\,bi,i}\right]\cdot V_i &= k_{on,\,mono}\cdot\alpha_i\cdot Ag_i\cdot AgAb^{*}_{fa\,mono,i}-2\cdot k_{off}\cdot AgAb^{*}_{fa\,bi,\,i}\\&\quad-\lambda_{db}\cdot AgAb^{*}_{fa\,bi,\,i}-\lambda_{phy}\cdot AgAb^{*}_{fa\,bi,\,i}\end{aligned}\tag{22}$$

Monovalent compartments

$$\begin{aligned}\frac{d}{dt}\left[AgAb_{fa\,mono,i}\right]\cdot V_i &= 2\cdot k_{on,\,mono}\cdot Ag_i\cdot\frac{Ab_{fa\,i}}{V_i}-k_{on,\,mono}\cdot\alpha_i\cdot Ag_i\cdot AgAb_{fa\,mono,i}\\&\quad-k_{off}\cdot AgAb_{fa\,mono,i}+2\cdot k_{off}\cdot AgAb_{fa\,bi,i}-\lambda_{db}\cdot AgAb_{fa\,mono,i}+\lambda_{phy}\cdot AgAb^{*}_{fa\,mono,\,i}\\\frac{d}{dt}\left[AgAb^{*}_{fa\,mono,i}\right]\cdot V_i &= 2\cdot k_{on,\,mono}\cdot Ag_i\cdot\frac{Ab^{*}_{fa\,i}}{V_i}-k_{on,\,mono}\cdot\alpha_i\cdot Ag_i\cdot AgAb^{*}_{fa\,mono,\,i}\\&\quad-k_{off}\cdot AgAb^{*}_{fa\,mono,\,i}+2\cdot k_{off}\cdot AgAb^{*}_{fa\,bi,i}-\lambda_{db}\cdot AgAb^{*}_{fa\,mono,\,i}-\lambda_{phy}\cdot AgAb^{*}_{fa\,mono,\,i}\end{aligned}\tag{23}$$

Free antibody (i = red marrow, spleen):

$$\begin{aligned}\frac{d}{dt}\left[Ab_{fa\,i}\right]\cdot V_i &= -2\cdot k_{on,\,mono}\cdot Ag_i\cdot\frac{Ab_{fa\,i}}{V_i}+k_{off}\cdot AgAb_{fa\,mono,i}+\frac{F_i}{V_P}\cdot Ab_{fa\,P}\\&\quad-\frac{F_i}{V_i}\cdot Ab_{fa\,i}+\lambda_{phy}\cdot Ab^{*}_{fa\,i}\\\frac{d}{dt}\left[Ab^{*}_{fa\,i}\right]\cdot V_i &= -2\cdot k_{on,\,mono}\cdot Ag_i\cdot\frac{Ab^{*}_{fa\,i}}{V_i}+k_{off}\cdot AgAb^{*}_{fa\,mono,\mathrm{i}}+\frac{F_i}{V_P}\cdot Ab^{*}_{fa\,P}\\&\quad-\frac{F_i}{V_i}\cdot Ab^{*}_{fa\,i}-\lambda_{phy}\cdot Ab^{*}_{fa\,i}\end{aligned}\tag{24}$$

Free antibody liver

$$\begin{aligned}\frac{d}{dt}\left[Ab_{fa\,L}\right]\cdot V_L &= -2\cdot k_{on,\,mono}\cdot Ag_L\cdot\frac{Ab_{fa\,L}}{V_L}+k_{off}\cdot AgAb_{fa\,mono,L}+\frac{F_L}{V_P}\cdot Ab_{fa\,P}+\frac{F_S}{V_S}\cdot Ab_{fa\,S}\\&\quad+\frac{F_{GI}}{V_{\mathrm{GI}}}\cdot Ab_{fa\,GI}-\frac{F_L+F_S+F_{GI}}{V_L}\cdot Ab_{fa\,L}+\lambda_{phy}\cdot Ab^{*}_{fa\,L}\\\frac{d}{dt}\left[Ab^{*}_{fa\,L}\right]\cdot V_L &= -2\cdot k_{on,\,mono}\cdot Ag_L\cdot\frac{Ab^{*}_{fa\,L}}{V_L}+k_{off}\cdot AgAb^{*}_{fa\,mono,\mathrm{L}}+\frac{F_L}{V_P}\cdot Ab^{*}_{fa\,P}+\frac{F_S}{V_S}\cdot Ab^{*}_{fa\,S}\\&\quad+\frac{F_{GI}}{V_{\mathrm{GI}}}\cdot Ab^{*}_{fa\,GI}-\frac{F_L+F_S+F_{GI}}{V_L}\cdot Ab^{*}_{fa\,L}-\lambda_{phy}\cdot Ab^{*}_{fa\,L}\end{aligned}\tag{25}$$

Free antibody gastrointestinal tract

$$\frac{d}{dt}\left[Ab_{fa\,GI}\right]\cdot V_{GI} = \frac{F_{GI}}{V_P}\cdot Ab_{fa\,P} - \frac{F_{GI}}{V_{GI}}\cdot Ab_{fa\,GI} + \lambda_{phy}\cdot Ab^*_{fa\,GI}$$
$$\frac{d}{dt}\left[Ab^*_{fa\,GI}\right]\cdot V_{GI} = \frac{F_{GI}}{V_P}\cdot Ab^*_{fa\,P} - \frac{F_{GI}}{V_{GI}}\cdot Ab^*_{fa\,GI} - \lambda_{phy}\cdot Ab^*_{fa\,GI} \quad (26)$$

Free antibody interstitial

$$\frac{d}{dt}Ab_{fa\,\mathrm{int}} = k_{in}\cdot Ab_{fa\,P} - k_{out}\cdot Ab_{fa\,\mathrm{int}} + \lambda_{phy}\cdot Ab^*_{fa\,\mathrm{int}}$$
$$\frac{d}{dt}Ab^*_{fa\,\mathrm{int}} = k_{in}\cdot Ab^*_{fa\,P} - k_{out}\cdot Ab^*_{fa\,\mathrm{int}} - \lambda_{phy}\cdot Ab^*_{fa\,\mathrm{int}} \quad (27)$$

Free main vascular compartment

$$\frac{d}{dt}\left[Ab_{fa\,P}\right]\cdot V_P = -2\cdot k_{on,\,mono}\cdot Ag_P\cdot\frac{Ab_{fa\,P}}{V_P} + k_{off}\cdot AgAb_{fa\,mono,P} - \frac{F_L+F_S+F_{GI}+F_{RM}}{V_P}\cdot Ab_{fa\,P}$$
$$+\frac{F_L+F_S+F_{GI}}{V_L}\cdot Ab_{fa\,L} + \frac{F_{RM}}{V_{RM}}\cdot Ab_{fa\,RM} - (\lambda_{du}+k_{in})\cdot Ab_{fa\,P} + k_{out}\cdot Ab_{fa\,\mathrm{int}} + \lambda_{phy}\cdot Ab^*_{fa\,P} \quad (28)$$
$$\frac{d}{dt}\left[Ab^*_{fa\,P}\right]\cdot V_P = -2\cdot k_{on,\,mono}\cdot Ag_P\cdot\frac{Ab^*_{fa\,P}}{V_P} + k_{off}\cdot AgAb^*_{fa\,mono,P} - \frac{F_L+F_S+F_{GI}+F_{RM}}{V_P}\cdot Ab^*_{fa\,P}$$
$$+\frac{F_L+F_S+F_{GI}}{V_L}\cdot Ab^*_{fa\,L} + \frac{F_{RM}}{V_{RM}}\cdot Ab^*_{fa\,RM} - (\lambda_{du}+k_{in}+\lambda_{phy})\cdot Ab^*_{fa\,P} + k_{out}\cdot Ab^*_{fa\,\mathrm{int}}$$

Description of global model: Part B, half immunoreactive antibody

Monovalent compartments

$$\frac{d}{dt}\left[AgAb_{ha\,mono,i}\right]\cdot V_i = 2\cdot k_{on,\,mono}\cdot Ag_i\cdot\frac{Ab_{ha\,i}}{V_i} - k_{on,\,mono}\cdot\alpha_i\cdot Ag_i\cdot AgAb_{ha\,mono,i} - k_{off}\cdot AgAb_{ha\,mono,i} + 2\cdot k_{off}\cdot AgAb_{ha\,bi,i} - \lambda_{db}\cdot AgAb_{ha\,mono,i} + \lambda_{phy}\cdot AgAb^*_{ha\,mono,\,i}$$

$$\frac{d}{dt}\left[AgAb^*_{ha\,mono,i}\right]\cdot V_i = 2\cdot k_{on,\,mono}\cdot Ag_i\cdot\frac{Ab^*_{ha\,i}}{V_i} - k_{on,\,mono}\cdot\alpha_i\cdot Ag_i\cdot AgAb^*_{ha\,mono,\,i} - k_{off}\cdot AgAb^*_{ha\,mono,\,i} + 2\cdot k_{off}\cdot AgAb^*_{ha\,bi,i} - \lambda_{db}\cdot AgAb^*_{ha\,mono,\,i} - \lambda_{phy}\cdot AgAb^*_{ha\,mono,\,i} \quad (29)$$

Free antibody (i = red marrow, spleen):

$$\frac{d}{dt}\left[Ab_{ha\,i}\right]\cdot V_i = -2\cdot k_{on,\,mono}\cdot Ag_i\cdot\frac{Ab_{ha\,i}}{V_i} + k_{off}\cdot AgAb_{ha\,mono,i} + \frac{F_i}{V_P}\cdot Ab_{ha\,P} - \frac{F_i}{V_i}\cdot Ab_{ha\,i} + \lambda_{phy}\cdot Ab^*_{ha\,i}$$

$$\frac{d}{dt}\left[Ab^*_{ha\,i}\right]\cdot V_i = -2\cdot k_{on,\,mono}\cdot Ag_i\cdot\frac{Ab^*_{ha\,i}}{V_i} + k_{off}\cdot AgAb^*_{ha\,mono,\mathrm{i}} + \frac{F_i}{V_P}\cdot Ab^*_{ha\,P} - \frac{F_i}{V_i}\cdot Ab^*_{ha\,i} - \lambda_{phy}\cdot Ab^*_{ha\,i} \quad (30)$$

Free antibody liver

$$\frac{d}{dt}\left[Ab_{ha\,L}\right]\cdot V_L = -2\cdot k_{on,\,mono}\cdot Ag_L\cdot\frac{Ab_{ha\,L}}{V_L} + k_{off}\cdot AgAb_{ha\,mono,\,L} + \frac{F_L}{V_P}\cdot Ab_{ha\,P} + \frac{F_S}{V_S}\cdot Ab_{ha\,S} + \frac{F_{\mathrm{GI}}}{V_{\mathrm{GI}}}\cdot Ab_{ha\,GI} - \frac{F_L+F_S+F_{GI}}{V_L}\cdot Ab_{ha\,L} + \lambda_{phy}\cdot Ab^*_{ha\,L}$$

$$\frac{d}{dt}\left[Ab^*_{ha\,L}\right]\cdot V_L = -2\cdot k_{on,\,mono}\cdot Ag_L\cdot\frac{Ab^*_{ha\,L}}{V_L} + k_{off}\cdot AgAb^*_{ha\,mono,\mathrm{L}} + \frac{F_L}{V_P}\cdot Ab^*_{ha\,P} + \frac{F_S}{V_S}\cdot Ab^*_{ha\,S} + \frac{F_{\mathrm{GI}}}{V_{\mathrm{GI}}}\cdot Ab^*_{ha\,GI} - \frac{F_L+F_S+F_{GI}}{V_L}\cdot Ab^*_{ha\,L} - \lambda_{phy}\cdot Ab^*_{ha\,L} \quad (31)$$

Free antibody gastrointestinal tract

$$\begin{aligned}
\frac{d}{dt}\left[Ab_{ha\,GI}\right]\cdot V_{GI} &= \frac{F_{GI}}{V_P}\cdot Ab_{ha\,P} - \frac{F_{GI}}{V_{GI}}\cdot Ab_{ha\,GI} + \lambda_{phy}\cdot Ab^{*}_{ha\,GI}\\
\frac{d}{dt}\left[Ab^{*}_{ha\,GI}\right]\cdot V_{GI} &= \frac{F_{GI}}{V_P}\cdot Ab^{*}_{ha\,P} - \frac{F_{GI}}{V_{GI}}\cdot Ab^{*}_{ha\,GI} - \lambda_{phy}\cdot Ab^{*}_{ha\,GI}
\end{aligned} \tag{32}$$

Free antibody interstitial

$$\begin{aligned}
\frac{d}{dt}Ab_{ha\,\mathrm{int}} &= k_{in}\cdot Ab_{ha\,P} - k_{out}\cdot Ab_{ha\,\mathrm{int}} + \lambda_{phy}\cdot Ab^{*}_{ha\,\mathrm{int}}\\
\frac{d}{dt}Ab^{*}_{ha\,\mathrm{int}} &= k_{in}\cdot Ab^{*}_{ha\,P} - k_{out}\cdot Ab^{*}_{ha\,\mathrm{int}} - \lambda_{phy}\cdot Ab^{*}_{ha\,\mathrm{int}}
\end{aligned} \tag{33}$$

Free main vascular compartment

$$\begin{aligned}
\frac{d}{dt}\left[Ab_{ha\,P}\right]\cdot V_P &= -2\cdot k_{on,\,mono}\cdot Ag_P\cdot\frac{Ab_{ha\,P}}{V_P} + k_{off}\cdot AgAb_{ha\,mono,P} - \frac{F_L+F_S+F_{GI}+F_{RM}}{V_P}\cdot Ab_{ha\,P}\\
&\quad + \frac{F_L+F_S+F_{GI}}{V_L}\cdot Ab_{ha\,L} + \frac{F_{RM}}{V_{RM}}\cdot Ab_{ha\,RM} - (\lambda_{du}+k_{in})\cdot Ab_{ha\,P} + k_{out}\cdot Ab_{ha\,\mathrm{int}} + \lambda_{phy}\cdot Ab^{*}_{ha\,P}\\
\frac{d}{dt}\left[Ab^{*}_{ha\,P}\right]\cdot V_P &= -2\cdot k_{on,\,mono}\cdot Ag_P\cdot\frac{Ab^{*}_{ha\,P}}{V_P} + k_{off}\cdot AgAb^{*}_{ha\,mono,P} - \frac{F_L+F_S+F_{GI}+F_{RM}}{V_P}\cdot Ab^{*}_{ha\,P}\\
&\quad + \frac{F_L+F_S+F_{GI}}{V_L}\cdot Ab^{*}_{ha\,L} + \frac{F_{RM}}{V_{RM}}\cdot Ab^{*}_{ha\,RM} - (\lambda_{du}+k_{in}+\lambda_{phy})\cdot Ab^{*}_{ha\,P} + k_{out}\cdot Ab^{*}_{ha\,\mathrm{int}}
\end{aligned} \tag{34}$$

Description of global model: Part C, none immunoreactive antibody

Free antibody (i = red marrow, spleen):

$$\begin{aligned}\frac{d}{dt}\left[Ab_{na\,i}\right]\cdot V_i &= -\frac{F_i}{V_P}\cdot Ab_{na\,P} - \frac{F_i}{V_i}\cdot Ab_{na\,i} + \lambda_{phy}\cdot Ab^*_{na\,i}\\ \frac{d}{dt}\left[Ab^*_{na\,i}\right]\cdot V_i &= +\frac{F_i}{V_P}\cdot Ab^*_{na\,P} - \frac{F_i}{V_i}\cdot Ab^*_{na\,i} - \lambda_{phy}\cdot Ab^*_{na\,i}\end{aligned} \quad (35)$$

Free antibody liver

$$\begin{aligned}\frac{d}{dt}\left[Ab_{na\,L}\right]\cdot V_L &= +\frac{F_L}{V_P}\cdot Ab_{na\,P} + \frac{F_S}{V_S}\cdot Ab_{na\,S} + \frac{F_{GI}}{V_{\mathrm{GI}}}\cdot Ab_{na\,GI} - \frac{F_L+F_S+F_{GI}}{V_L}\cdot Ab_{na\,L} + \lambda_{phy}\cdot Ab^*_{na\,L}\\ \frac{d}{dt}\left[Ab^*_{na\,L}\right]\cdot V_L &= +\frac{F_L}{V_P}\cdot Ab^*_{na\,P} + \frac{F_S}{V_S}\cdot Ab^*_{na\,S} + \frac{F_{GI}}{V_{\mathrm{GI}}}\cdot Ab^*_{na\,GI} - \frac{F_L+F_S+F_{GI}}{V_L}\cdot Ab^*_{na\,L} - \lambda_{phy}\cdot Ab^*_{na\,L}\end{aligned} \quad (36)$$

Free antibody gastrointestinal tract

$$\begin{aligned}\frac{d}{dt}\left[Ab_{na\,GI}\right]\cdot V_{GI} &= \frac{F_{GI}}{V_P}\cdot Ab_{na\,P} - \frac{F_{GI}}{V_{GI}}\cdot Ab_{na\,GI} + \lambda_{phy}\cdot Ab^*_{na\,GI}\\ \frac{d}{dt}\left[Ab^*_{na\,GI}\right]\cdot V_{GI} &= \frac{F_{GI}}{V_P}\cdot Ab^*_{na\,P} - \frac{F_{GI}}{V_{GI}}\cdot Ab^*_{na\,GI} - \lambda_{phy}\cdot Ab^*_{na\,GI}\end{aligned} \quad (37)$$

Free antibody interstitial

$$\begin{aligned}\frac{d}{dt}Ab_{na\,\mathrm{int}} &= k_{in}\cdot Ab_{na\,P} - k_{out}\cdot Ab_{na\,\mathrm{int}} + \lambda_{phy}\cdot Ab^*_{na\,\mathrm{int}}\\ \frac{d}{dt}Ab^*_{na\,\mathrm{int}} &= k_{in}\cdot Ab^*_{na\,P} - k_{out}\cdot Ab^*_{na\,\mathrm{int}} - \lambda_{phy}\cdot Ab^*_{na\,\mathrm{int}}\end{aligned} \quad (38)$$

Free main vascular compartment

$$\frac{d}{dt}\left[Ab_{na\,P}\right]\cdot V_P = -\frac{F_L + F_S + F_{GI} + F_{RM}}{V_P}\cdot Ab_{na\,P} + \frac{F_L + F_S + F_{GI}}{V_L}\cdot Ab_{na\,L} + \frac{F_{RM}}{V_{RM}}\cdot Ab_{na\,RM} - (\lambda_{du} + k_{in})\cdot Ab_{na\,P} + k_{out}\cdot Ab_{ha\,\mathrm{int}} + \lambda_{phy}\cdot Ab^*_{ha\,P}$$

$$\frac{d}{dt}\left[Ab^*_{na\,P}\right]\cdot V_P = -\frac{F_L + F_S + F_{GI} + F_{RM}}{V_P}\cdot Ab^*_{na\,P} + \frac{F_L + F_S + F_{GI}}{V_L}\cdot Ab^*_{na\,L} + \frac{F_{RM}}{V_{RM}}\cdot Ab^*_{na\,RM} - (\lambda_{du} + k_{in} + \lambda_{phy})\cdot Ab^*_{na\,P} + k_{out}\cdot Ab^*_{na\,\mathrm{int}} \qquad (39)$$

Submodel (for j=fa, ha, na)

The following sub model for degraded antibody (^{111}In-DTPA and ^{111}In-low molecular weight biodistribution) was adopted from Eger et al. [1]. The variables are defined in Table A.

$$\frac{d}{dt}Ex_j = \lambda_{du}\cdot Ab_P - \lambda_{clex}\cdot Ex_j + \lambda_{phy}\cdot Ex_j^* \qquad (40)$$

$$\frac{d}{dt}Ex_j^* = \lambda_{du}\cdot Ab_P^* - \lambda_{clex}\cdot Ex_j^* - \lambda_{phy}\cdot Ex_j^*$$

$$\frac{d}{dt}Meta_{P,j} = \lambda_{clex}\cdot Ex_j - \lambda_{cl}\cdot Meta_{P,j} - \lambda_{Metaex1}\cdot Meta_{P,j} + \lambda_{Metaex2}\cdot Meta_{ex1,j} + \lambda_{db}\cdot\sum_i (AgAb_{mono,i} + AgAb_{\mathrm{bi},i}) + \lambda_{phy}\cdot Meta^*_{P,j}$$

$$\frac{d}{dt}Meta^*_{P,j} = \lambda_{clex}\cdot Ex_j^* - \lambda_{cl}\cdot Meta^*_{P,j} - \lambda_{Metaex1}\cdot Meta^*_{P,j} + \lambda_{Metaex2}\cdot Meta^*_{ex1,j} + \lambda_{db}\cdot\sum_i (AgAb^*_{mono,i} + AgAb^*_{\mathrm{bi},i}) - \lambda_{phy}\cdot Meta^*_{P,j} \qquad (41)$$

$$\begin{aligned}
\frac{d}{dt} Meta_{ex1,j} &= \lambda_{Metaex1} \cdot Meta_{P,j} - \left(\lambda_{Metaex2} + \lambda_{Metaex3}\right) \cdot Meta_{ex1,j} \\
&\quad + \lambda_{Metaex4} \cdot Meta_{ex2,j} + \lambda_{phy} \cdot Meta^{*}_{ex1,j} \\
\frac{d}{dt} Meta^{*}_{ex1,j} &= \lambda_{Metaex1} \cdot Meta^{*}_{P,j} - \left(\lambda_{Metaex2} + \lambda_{Metaex3}\right) \cdot Meta^{*}_{ex1,j} \\
&\quad + \lambda_{Metaex4} \cdot Meta^{*}_{ex2,j} - \lambda_{phy} \cdot Meta^{*}_{ex1,j}
\end{aligned} \tag{42}$$

$$\begin{aligned}
\frac{d}{dt} Meta_{ex2,j} &= \lambda_{Metaex3} \cdot Meta_{ex1,j} - \lambda_{Metaex4} \cdot Meta_{ex2,j} + \lambda_{phy} \cdot Meta^{*}_{ex2,j} \\
\frac{d}{dt} Meta^{*}_{ex2,j} &= \lambda_{Metaex3} \cdot Meta^{*}_{ex1,j} - \lambda_{Metaex4} \cdot Meta^{*}_{ex2,j} - \lambda_{phy} \cdot Meta^{*}_{ex2,j}
\end{aligned} \tag{43}$$

TABLE B Parameter definition

Variable	Description	Value	Unit	Source
r_{im}	probability for an antibody arm to be immunoreactive	estimated	unity	
$k_{on,\ mono}$	association rate monovalent	0.006	$l\cdot nmol^{-1}\cdot min^{-1}$	[3]
$k_{on,\ bi}$	surface association rate bivalent	$k_{on,\ bi} = k_{on,\ mono} \cdot E$	$cm^2\cdot nmol^1 \cdot min^1$	[6]
k_{off}	dissociation rate	0.06	min^{-1}	[12]
α_i	abbreviation of enhancement factor and surface area of all cells of organ i	$E / (4 \cdot \pi \cdot r_{cell}{}^2 N_{cells,\ i})$	cm^{-3}	[6]
E	enhancement factor	1672000	cm^{-1}	[6]
BW	body weight	individually measured	kg	
$N_{cell,\ RM}$	no. of CD66 expressing cells in the red m.	$188\cdot 10^8$/kg·BW	unity	[7,13]
$N_{cell,\ S,m}$	no. of marginating granulocytes in the spleen	$N_{cell,\ S,m} = Ag_{0,S}/Ag_{0,KM}\cdot N_{cell,\ RM}$	unity	[14]
$N_{cell,\ L,m}$	no. of marginating granulocytes in the liver	$N_{cell,\ L,m} = Ag_{0,L}/Ag_{0,KM}\cdot N_{cell,\ RM}$	unity	[14]
$N_{cell,\ B,c}$	no. of circulating granulocytes in blood	Equations (5),(6) and (7)	unity	[7,14]
$N_{cell,\ B,m}$	no. of marginating granulocytes in blood	Equations (5),(6) and (7)	unity	[7,14]
r_{cell}	cell radius	6.0	µm	[15]
F	total plasma flow	V·1.23*	$ml\cdot min^{-1}$	
F_L	plasma flow liver arterial	F·0.065	$ml\cdot min^{-1}$	[5]
F_S	plasma flow to spleen	F·0.03	$ml\cdot min^{-1}$	[5]
F_{GI}	plasma flow to GI tract	0.16·F	$ml\cdot min^{-1}$	[5]
F_{RM}	plasma flow to red marrow	$F\cdot f_{rm}$	$ml\cdot min^{-1}$	
f_{rm}	fractional flow to red marrow	estimated	%	
V	total plasma volume	estimated with Bayesian information†	ml	
$V_{MRI,L}$	measured total liver volume	measured individually	ml	
$V_{MRI,S}$	measured total spleen volume	measured individually	ml	

$V_{Cal,L}$	calculated total liver volume	calculated individually	ml	[10]
$V_{Cal,S}$	calculated total spleen volume	calculated individually	ml	[9]
$V_{ROI,L2-L4}$	blood volume in red marrow ROI from overlapping arteries and veins	30	ml	[16]
V_L	volume of Ab distribution liver	V ·0.1	ml	
V_S	volume of Ab distribution spleen	V ·0.014	ml	
V_{GI}	volume of Ab distribution GI	0.076·V	ml	[5]
V_{RM}	volume of Ab distribution red marrow	0.04·V	ml	[5]
V_P	total plasma minus organs	$V - V_L - V_S - V_{GI} - V_{RM}$	ml	
λ_{phy}	physical decay ^{111}In and ^{90}Y	$1.72 \cdot 10^{-4}$ and $1.80 \cdot 10^{-4}$	min^{-1}	
k_{in}	transport rate V_P to interstitial space	0.0017	min^{-1}	[1]
k_{out}	transport rate interstitial space to V_P	0.005	min^{-1}	[1]
λ_{db}	degradation of bound antibody	estimated	min^{-1}	
λ_{du}	degradation of unbound antibody	$3.9 \cdot 10^{-4}$	min^{-1}	[1]
Ab	antibody			
PTM	pre-therapeutic measurements			
a	radiochemical purity PTM	0.94±0.03 measured	unity	
b	radiochemical purity therapy	0.96±0.03 measured	unity	
$f_{l,PT}$	fraction of labeled antibody PTM	2.3±0.3	%	
$f_{l,T}$	fraction of labeled antibody therapy	21±6	%	
$Ab_{total,PT}$	total amount of antibody used for labeling PTM			
$Ab_{total,T}$	total amount of antibody used for labeling therapy			
$A_{total,T}$	total amount of activity used for labeling PTM			
$A_{total,PT}$	total amount of activity used for labeling therapy			

$A_{inj,PT}$	injected activity PTM			
$A_{inj,T}$	injected activity therapy			
$Ab^*_{inj,PT}$	total amount of radiolabeled injected Ab for PTM		nmol	
$Ab_{inj,PT}$	total amount of unlabeled injected Ab for PTM		nmol	
$Ab^*_{inj,T}$	total amount of radiolabeled injected Ab for therapy		nmol	
$Ab_{inj,T}$	total amount of unlabeled injected Ab for therapy		nmol	
$Ab^*_{fa,inj,PT}$	radiolabeled injected fully immunoreactive Ab for PTM		nmol	
$Ab^*_{fa,inj,T}$	radiolabeled injected fully immunoreactive Ab for therapy		nmol	
$Ab^*_{ha,inj,PT}$	radiolabeled injected half immunoreactive Ab for PTM		nmol	
$Ab^*_{ha,inj,T}$	radiolabeled injected half immunoreactive Ab for therapy		nmol	
$Ab^*_{na,inj,PT}$	radiolabeled injected none immunoreactive Ab for PTM		nmol	
$Ab^*_{na,inj,T}$	radiolabeled injected none immunoreactive Ab for therapy		nmol	
$Ab_{fa,inj,PT}$	unlabeled injected fully immunoreactive Ab for PTM		nmol	
$Ab_{fa,inj,T}$	unlabeled injected fully immunoreactive Ab for therapy		nmol	
$Ab_{ha,inj,PT}$	unlabeled injected half immunoreactive Ab for PTM		nmol	
$Ab_{ha,inj,T}$	unlabeled injected half immunoreactive Ab for therapy		nmol	
$Ab_{na,inj,PT}$	unlabeled injected none immunoreactive Ab for PTM		nmol	
$Ab_{na,inj,T}$	unlabeled injected none immunoreactive Ab for therapy		nmol	
$Ab_{fa,i}$	unbound fully immunoreactive Ab in organ i		nmol	
$Ab_{ha,i}$	unbound half immunoreactive Ab in organ i		nmol	
$Ab_{na,i}$	unbound none immunoreactive Ab in organ i		nmol	
$Ab_{\text{int},j}$	unbound antibody of interstitial spaces		nmol	
$AgAb_{fa\,\text{bi},i}$	bivalently bound fully immunoreactive Ab of organ i		nmol	
$AgAb_{fa\,\text{mono},i}$	monovalently bound fully immunoreactive Ab of organ i		nmol	
$AgAb_{ha\,\text{mono},i}$	monovalently bound half immunoreactive Ab of organ i		nmol	
R_{PT}	free nuclides for PTM		nmol	
R_T	free nuclides for therapy		nmol	
Ag_i	number of free antigens of organ i		nmol	

$[Ag]_s$	surface concentration of antigen		$mol \cdot cm^{-2}$	
$Ag_{0,i}$	total antigens of organ i	estimated	nmol	
Ex_j	degraded antibody in extra vascular (e.v.) delay		nmol	[1]
$Meta_{P,j}$	degraded antibody in plasma		nmol	[17]
$Meta_{ex1,j}$	degraded antibody in e.v. 1		nmol	[17]
$Meta_{ex2,j}$	degraded antibody in e.v. 2		nmol	[17]
λ_{clex}	clearance from e.v. delay space	$3.9 \cdot 10^{-5}$	min^{-1}	[1]
$\lambda_{Metaex1}$	vascular to e.v. 1	0.39	min^{-1}	[17]
$\lambda_{Metaex2}$	e.v. 1 to vascular	0.17	min^{-1}	[17]
$\lambda_{Metaex3}$	e.v. 1 to e.v. 2	0.018	min^{-1}	[17]
$\lambda_{Metaex4}$	e.v. 2 to e.v. 1	0.013	min^{-1}	[17]
λ_{cl}	clearance from body	0.047	min^{-1}	[17]

*For the average normal adult (blood) F = 6500 ml/min and V = 5300 ml. Therefore, a factor of 1.23 was assigned to account for the changes in total blood flow due to volume changes.

** These values were determined using the average values reported by Shah et al. [4]

† For the total serum volume V, a Bayesian term was derived from the first serum measurement $V_{m,1}$= 100%/ $[\%Activity]_{serum, 1}$ and the body surface area.[18] V was set to $V_{m,1}$ and the standard deviation of V to $V_{m,1}$ -V_{BSA}.

References

1. Eger RR, Covell DG, Carrasquillo JA, Abrams PG, Foon KA, et al. (1987) Kinetic model for the biodistribution of an 111In-labeled monoclonal antibody in humans. Cancer Res 47: 3328-3336.
2. Baxter LT, Zhu H, Mackensen DG, Butler WF, Jain RK (1995) Biodistribution of Monoclonal Antibodies: Scale-up Mouse to Human using a Physiologically Based Pharmacokinetic Model. Cancer Res 55: 4611-4622.
3. Graff CP, Wittrup KD (2003) Theoretical analysis of antibody targeting of tumor spheroids: importance of dosage for penetration, and affinity for retention. Cancer Res 63: 1288-1296.
4. Shah DK, Betts AM (2012) Towards a platform PBPK model to characterize the plasma and tissue disposition of monoclonal antibodies in preclinical species and human. J Pharmacokinet Pharmacodyn 39: 67-86.
5. Leggett RW, Williams LR (1995) A proposed blood circulation model for reference man. Health Phys 69: 187-201.
6. Kaufman EN, Jain RK (1992) Effect of Bivalent Interaction upon Apparent Antibody Affinity: Experimental Confirmation of Theory Using Fluorescence Photobleaching and Implications for Antibody Binding Assays. Cancer Res 52: 4157-4167.
7. Donohue DM, Reiff RH, Hanson ML, Betson Y, Finch CA (1958) Quantitative measurement of the erythrocytic and granulocytic cells of the marrow and blood. J Clin Invest 37: 1571-1576.
8. Peters AM, Saverymuttu SH, Bell RN, Lavender JP (1985) Quantification of the distribution of the marginating granulocyte pool in man. Scand J Haematol 34: 111-120.
9. Harris A, Kamishima T, Hao HY, Kato F, Omatsu T, et al. (2010) Splenic volume measurements on computed tomography utilizing automatically contouring software and its relationship with age, gender, and anthropometric parameters. Eur J Radiol 75: e97-101.
10. Johnson TN, Tucker GT, Tanner MS, Rostami-Hodjegan A (2005) Changes in liver volume from birth to adulthood: a meta-analysis. Liver Transpl 11: 1481-1493.
11. Glatting G, Landmann M, Kull T, Wunderlich A, Blumstein NM, et al. (2005) Internal radionuclide therapy: The UlmDos software for treatment planning. Med Phys 32: 2399-2405.
12. Johnstone RW, Andrew SM, Hogarth MP, Pietersz GA, McKenzie IFC (1990) The effect of temperature on the binding kinetics and equilibrium constants of monocolonal antibodies to cell surface antigens. Mol Immunol 27: 327 - 333.
13. Cartwright GE, Athens JW, Wintrobe MM (1964) Analytical Review: The Kinetics of Granulopoiesis in Normal Man. Blood.
14. Peters AM, Saverymuttu SH, Keshavarzian A, Bell RN, Lavender JP (1985) Splenic pooling of granulocytes. Clin Sci 68: 283-289.
15. Griffith AW, Cooper JM (1998) Single-Cell Measurements of Human Neutrophil Activation Using Electrorotation. Anal Chem 70: 2607-2612.
16. Meredith RF, Shen S, Forero A, Lobuglio A (2008) A method to correct for radioactivity in large vessels that overlap the spine in imaging-based marrow dosimetry of lumbar vertebrae. J Nucl Med 49: 279-284.
17. Houston AS, Sampson WF, Macleod MA (1979) A compartmental model for the distribution of 113mIn-DTPA and 99mTc-(Sn)DTPA in man following intravenous injection. Int J Nucl Med Biol 6: 85-95.
18. Buchmann I, Kull T, Glatting G, Bunjes D, Hale G, et al. (2003) A comparison of the biodistribution and biokinetics of 99mTc-anti-CD66 mAb BW 250/183 and 99mTc-anti-CD45 mAb YTH 24.5 with regard to suitability for myeloablative radioimmunotherapy. Eur J Nucl Med Mol Imaging 30: 667-673.
